# Enhanced C‐To‐T and A‐To‐G Base Editing in Mitochondrial DNA with Engineered DdCBE and TALED

**DOI:** 10.1002/advs.202304113

**Published:** 2023-11-20

**Authors:** Yinghui Wei, Ming Jin, Shuhong Huang, Fangyao Yao, Ningxin Ren, Kun Xu, Shangpu Li, Pengfei Gao, Yingsi Zhou, Yulin Chen, Hui Yang, Wen Li, Chunlong Xu, Meiling Zhang, Xiaolong Wang

**Affiliations:** ^1^ International Joint Agriculture Research Center for Animal Bio‐Breeding of Ministry of Agriculture and Rural Affairs College of Animal Science and Technology Northwest A&F University Yangling Shaanxi 712100 China; ^2^ School of Future Technology on Bio‐Breeding College of Animal Science and Technology Northwest A&F University Yangling Shaanxi 712100 China; ^3^ Department of Neurology and Institute of Neurology of First Affiliated Hospital Institute of Neuroscience, and Fujian Key Laboratory of Molecular Neurology Fujian Medical University Fuzhou Fujian 350004 China; ^4^ HuidaGene Therapeutics Co., Ltd. Shanghai 200131 China; ^5^ Shanghai Center for Brain Science and Brain‐Inspired Intelligence Shanghai 201602 China; ^6^ International Peace Maternity and Child Health Hospital School of Medicine Shanghai Jiao Tong University Shanghai 200030 China

**Keywords:** DdCBE, disease modeling, mitochondrial base editing, TALED

## Abstract

Mitochondrial base editing with DddA‐derived cytosine base editor (DdCBE) is limited in the accessible target sequences and modest activity. Here, the optimized DdCBE tools is presented with improved editing activity and expanded C‐to‐T targeting scope by fusing DddA11 variant with different cytosine deaminases with single‐strand DNA activity. Compared to previous DdCBE based on DddA11 variant alone, fusion of the activation‐induced cytidine deaminase (AID) from *Xenopus laevis* not only permits cytosine editing of 5′‐GC‐3′ sequence, but also elevates editing efficiency at 5′‐TC‐3′, 5′‐CC‐3′, and 5′‐GC‐3′ targets by up to 25‐, 10‐, and 6‐fold, respectively. Furthermore, the A‐to‐G editing efficiency is significantly improved by fusing the evolved DddA6 variant with TALE‐linked deoxyadenosine deaminase (TALED). Notably, the authors introduce the reported high‐fidelity mutations in DddA and add nuclear export signal (NES) sequences in DdCBE and TALED to reduce off‐target editing in the nuclear and mitochondrial genome while improving on‐target editing efficiency in mitochondrial DNA (mtDNA). Finally, these engineered mitochondrial base editors are shown to be efficient in installing mtDNA mutations in human cells or mouse embryos for disease modeling. Collectively, the study shows broad implications for the basic study and therapeutic applications of optimized DdCBE and TALED.

## Graphical abstract

The fusion of DddA11‐derived cytosine base editor with a single‐stranded deaminase (xAID), in conjunction with the nuclear export signal sequences (NESs), enables accurate C‐to‐T conversions with expanded target compatibility and enhanced activity in the mitochondrial genome. The authors also improve the efficiency and specificity of A‐to‐G editing by fusing the evolved DddA6 variant and NESs with TALE‐linked deoxyadenosine deaminase (TALED).

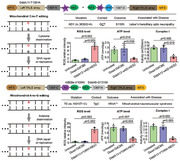

## 1 Introduction

Mutations in mitochondrial DNA (mtDNA) could result in severe diseases with different penetrance, presentation, and prognosis affecting at least 1 in 4300 individuals.^[^ ^1^, ^2^ ^]^ Tools for introducing specific modifications into mtDNA are urgently needed to both model and potentially treat these diseases. Regrettably, such precision editing has been held back by the inability to deliver guide RNAs of CRISPR‐Cas system with efficient nuclear gene editing activity into mitochondria matrix that is bound by the membrane with strong hydrophobicity and electrochemical potential.^[^ ^3^ ^]^ RNA‐free programmable nucleases, such as zinc finger nucleases^[^ ^4^, ^5^ ^]^ and transcription activator‐like effector nucleases (TALENs),^[^ ^6^, ^7^, ^8^, ^9^ ^]^ could be engineered to induce double‐strand breaks in mtDNA and then rapidly degrade the linearized mtDNA, resulting in heteroplasmic shifts to favor uncut mtDNA genomes. Specific nucleotide changes in mtDNA with homoplasmic mtDNA mutations could not be introduced by these approaches. Recently RNA‐free DddA‐derived cytosine base editors (DdCBEs)^[^ ^10^ ^]^ and transcription‐activator‐like effector (TALE)‐linked deaminases (TALEDs)^[^ ^11^ ^]^ have been developed to catalyze C‐to‐T and A‐to‐G editing in mtDNA. Given the strict sequence preference of DddA, initial DdCBE are largely limited to C‐to‐T conversions in the 5′‐TC‐3′ context.^[^ ^10^ ^]^ Mok et al. applied rapid phage‐assisted continuous evolution and related phage‐assisted non‐continuous evolution methods to develop two evolved DddA variants with improved activity at TC sequence context (DddA6) and expanded targeting scope at HC (H = A, C, or T) sequence context (DddA11).^[^ ^12^ ^]^ However, DdCBEs suitable for GC context targets are still unavailable. Recently, two studies successfully developed DddA orthology‐based cytosine base editors that can efficiently deaminate cytosine in GC context in mtDNA.^[^ ^13^, ^14^ ^]^ To solve the problem of inaccessible sequence compatibility, there are two commonly used methods in the field, one is to engineer the original deaminase, and the other is to find the new deaminase orthologs. Unlike strategies used in these two studies, our work mainly addressed this challenge by fusing the single‐strand activity of cytosine deaminase with DddA11.

We and others recently reported mitochondrial genome editing in human cells (embryos),^[^ ^10^, ^11^, ^12^, ^15^, ^16^, ^17^, ^18^ ^]^ animals,^[^ ^19^, ^20^, ^21^, ^22^, ^23^, ^24^ ^]^ plants^[^ ^25^, ^26^, ^27^, ^28^ ^]^ using DdCBEs, while causing substantial nuclear and mitochondrial off‐target mutations^[^ ^29^, ^30^, ^31^ ^]^ and raising concerns about the fidelity of DdCBEs. Lee et al. engineered high‐fidelity DddA‐derived cytosine base editors (HiFi‐DdCBEs) with minimal off‐target activity by substituting alanine for amino acid residues at the interface between the split DddA_tox_ halves.^[^ ^31^ ^]^ In this study, we fused the ssDNA deaminase (AID) from *Xenopus laevis* with DddA11 to generate optimized DdCBE exhibiting high activity and broad sequence compatibility. In addition, we developed highly efficient TALED tools by replacing wild‐type DddA with evolved DddA6 variant. Moreover, whole genome sequencing, whole mitochondrial genome sequencing, and targeted deep sequencing showed that we successfully improved the activity and specificity of these engineered DdCBE and TALED variants. Notably, we used these engineered DdCBEs and TALEDs to model disease‐associated mtDNA mutations that are inaccessible for previous mtDNA base editors, resulting in edited human cells presented with pathogenic phenotype.

## 2 Results

### 2.1 Addition of ssDNA Deaminases in DdCBE Increased Editing Efficiency and Accessible Sequences in mtDNA

**Figure 1:**
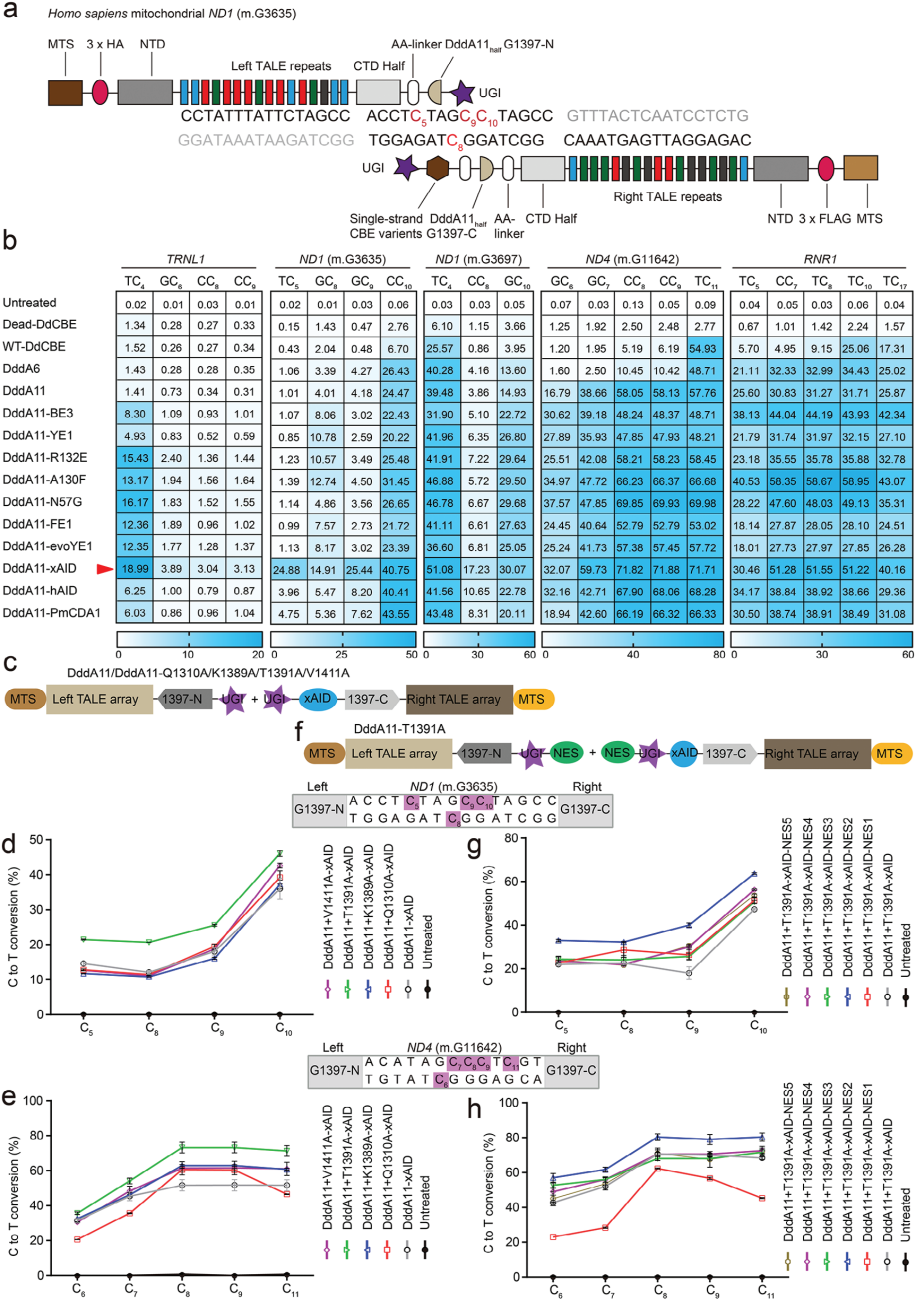
Engineered DdCBE variants show enhanced editing at TC, non‐TC target sequences in mtDNA. a) Schematic of engineered DdCBE variants targeting *ND1* (m.G3635) gene in the mitochondrial genome. b) Heat map showing C·G‐to‐T·A editing efficiencies induced by DddA (WT‐DdCBE), DddA6, DddA11, and DddA11‐fused cytosine deaminases with ssDNA activity in HEK293T cells at five mitochondrial target sites, including *TRNL1*, *ND1*, *ND4*, and *RNR1*. The cytosines in the top strands or bottom strands are presented as C‐to‐T conversion frequencies. The nucleotide adjacent to the end of the left‐TALE‐recognition sequence was numbered “‘1,”’ and C was sequentially numbered. For the heatmap, the number is given in units of %. Top 10% of EGFP‐ and mCherry‐double positive cells were harvested from FACS 48 h after transfection. The targeting efficiency was tested by targeted deep sequencing. c) Architectures of DddA11‐xAID with the reported high‐fidelity mutations. d,e) Analysis of C·G‐to‐T·A editing frequencies at *ND1* (m.G3635) and *ND4* (m.G11642) sites induced by DddA11‐xAID with high‐fidelity mutations, including Q1310A, K1389A, T1391A, and V1411A. f) Architectures of DddA11‐T1391A‐xAID fused different nuclear export signal (NES) sequences. g,h) Analysis of C·G‐to‐T·A editing frequencies at *ND1* (m.G3635) and *ND4* (m.G11642) sites induced by DddA11‐T1391A‐xAID fused NES sequences. Values and error bars in (b), (d,e), and (g,h) reflect the mean ± s.e.m. of *n* = 3 independent biological replicates.

To enable C‐to‐T editing for DdCBE in previously inaccessible 5′‐GC‐3′ targets, we investigated whether fusion of ssDNA deaminase to current TALE‐based DdCBE could enhance the activity of DddA, a dsDNA deaminase from the *bacterium Burkholderia cenocepacia*. Selected ssDNA deaminases are APOBEC1 variants (named BE3, YE1, R132E, FE1, and evoYE1), APOBEC3A variants (named A130F and N57G), AID (named hAID from *Homo sapiens* and xAID from *Xenopus laevis*), and PmCDA1 (Figure S1, Supporting Information). We designed DdCBE with customized TALE arrays targeting *ND1*, *ND4*, *TRNL1*, or *RNR1* gene in human mtDNA (**Figure** **1**a). DdCBE with dead DddA, wild‐type DddA (WT‐DdCBE),^[^ ^10^ ^]^ DddA6, and DddA11 variants^[^ ^12^ ^]^ were constructed as control. DddA halves are split at G1397 for all tested DdCBE variants. In HEK293T cells, we found that DddA11 actually increased average editing efficiencies at 5′‐TC‐3′ context. However, DddA11 yielded a relatively low editing efficiency at non‐TC target sites for four tested mitochondrial genes, especially at GC targets (Figure 1b). Targeted deep sequencing showed that the fused ssDNA deaminase with DddA11 (DddA11‐BE3, ‐YE1, ‐R132E, ‐A130F, ‐N57G, ‐FE1, ‐evoYE1, ‐hAID, ‐xAID, and ‐PmCDA1) was highly active, increasing C‐to‐T conversions across five mtDNA sites with average frequencies at TC (2.4‐fold), CC (2.1‐fold), and GC (1.8‐fold) compared to DdCBE with DddA11 alone, respectively (Figure 1b). Among ten variants, DddA11‐xAID supported the highest mtDNA editing efficiencies and most broad sequence activity, improving editing efficiencies at TC, CC, and GC targets for up to 24.6‐fold, 10.1‐fold, and 6.1‐fold in cell populations expressing both halves of dimeric DdCBE (Figure 1b).

To obtain potential high‐fidelity DdCBEs (HiFi‐DdCBEs) with minimal off‐target activity, we then proposed two different strategies (Figure 1c,f): 1) we introduced the reported high‐fidelity mutations (Q1310A,^[^ ^30^ ^]^ K1389A,^[^ ^31^ ^]^ T1391A,^[^ ^31^ ^]^ and V1411A^[^ ^31^ ^]^) in DddA to reduce the off‐target effect of DdCBE in the mitochondrial and nuclear genome; 2) we added different nuclear export signal (NES)^[^ ^18^, ^30^, ^32^ ^]^ sequences into the DdCBE architectures to further alleviate the nuclear off‐target effect by inhibiting the import of DdCBE to the nucleus. Previous study^[^ ^30^, ^31^ ^]^ reported that HiFi‐DdCBEs with interface mutations (Q1310A, K1389A, T1391A, and V1411A) in DddA or DddA11 largely avoided off‐target base editing. We therefore screened for high‐performance DdCBE candidates carrying these four mutations by editing two mtDNA loci. Compared with the DddA11‐xAID variant, engineered DdCBEs (DddA11‐T1391A‐xAID) induced the highest on‐target editing efficiencies at TC_5_ (14.59% to 21.50%), GC_8_ (12.10% to 20.70%), GC_9_ (18.08% to 25.57%), and CC_10_ (36.01% to 46.09%) for *ND1* (m.G3635) and at GC_7_ (45.38% to 54.14%), CC_8_ (51.66% to 73.23%) and TC_11_ (51.65% to 71.33%) for *ND4*, respectively (Figure 1d,e). Subsequently, fusing NES to the C terminus of DddA11‐T1391A also slightly enhanced on‐target mtDNA editing at *ND1* and *ND4* (m.G11642) sites in HEK293T cells. Incorporating NES2 into DddA11‐T1391A‐xAID resulted in a modest improvement of editing efficiency to 33.24%, 32.34%, 40.08%, and 63.67% at TC_5_, GC_8_, GC_9_, and CC_10_ targets for *ND1* (m.G3635) and 61.67%, 80.33%, and 81.02% at GC_7_, CC_8_, and TC_11_ targets for *ND4* (m.G11642) (Figure 1g,h).

In addition, we have checked the bystander edits in the *ND4*‐TALE binding sites and sites flanking the binding sites in the presence of cytosine deaminase (xAID). Our results demonstrated that 11NC (DddA11 + L‐1397N + R‐1397C) ‐T1391A‐xAID‐NES2 variant and WT‐DdCBE groups introduced slightly higher bystander edits in the TALE binding sites and sites flanking the binding sites compared to untreated group due to the presence of xAID (Figure S2a, Supporting Information). Moreover, we compared the bystander edits of our high‐activity variants with recently published mitoBEs,^[^ ^33^ ^]^ further confirming the result (Figure S2a, Supporting Information). These results collectively demonstrated that fusion ssDNA cytosine deaminase with DddA in an optimal DdCBE variant (DddA11‐T1391A‐xAID‐NES2) could improve the editing frequency and expand targeting scope for biological and therapeutic applications.

### 2.2 TALED with an Evolved DddA6 Variant Elevated A‐to‐G Editing Efficiency in mtDNA

**Figure 2:**
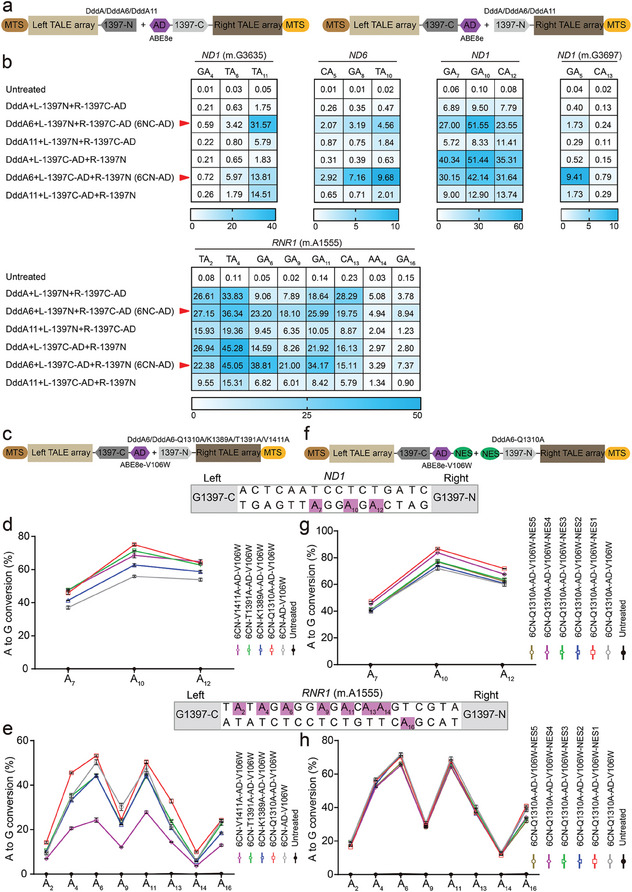
TALEDs fused evolved DddA6 achieve high‐efficiency editing at multiple mtDNA loci. a) Architectures of TALEDs with the evolved DddA6 or DddA11 variants. b) Heat map showing A·T‐to‐G·C editing efficiencies induced by TALED, TALED‐DddA6, and TALED‐DddA11 in HEK293T cells at five mitochondrial target sites, including *ND1*, *ND6*, and *RNR1*. TALEDs fused DddA6 variant and the split DddA_tox_ orientation at G1397 position that resulted in the higher editing efficiencies. The adenines in the top strands or bottom strands are presented as A‐to‐G conversion frequencies. The nucleotide adjacent to the end of the left‐TALE‐recognition sequence was numbered “‘1,”’ and A was sequentially numbered. For the heatmap, the number is given in units of %. Top 10% of EGFP‐ and mCherry‐double positive cells were harvested from FACS 48 h after transfection. The targeting efficiency was tested by targeted deep sequencing. c) Architectures of 6CN‐AD‐V106W with the reported high‐fidelity mutations of DddA. d,e) Analysis of A·T‐to‐G·C editing frequencies at *ND1* and *RNR1* (m.A1555) sites induced by 6CN‐AD‐V106W with high‐fidelity mutations, including Q1310A, K1389A, T1391A, and V1411A. f) Architectures of 6CN‐Q1310A‐AD‐V106W fused different nuclear export signal (NES) sequences. g,h) Analysis of A·T‐to‐G·C editing frequencies at *ND1* and *RNR1* (m.A1555) sites induced by 6CN‐Q1310A‐AD‐V106W fused NES sequences. For (b), (d,e), and (g,h), values and error bars reflect the mean ± s.e.m. of *n* = 3 independent biological replicates.

Recently, TALEDs have been developed to catalyze A‐to‐G conversions in human mitochondrial genome.^[^ ^11^ ^]^ However, we observed that the original TALEDs present relatively low editing efficiency at multiple mtDNA sites (**Figure** **2**b). It is worth noting that Mok et al. developed two evolved DddA variants (DddA6 and DddA11) with improved activity and expanded targeting scope. Therefore, we replaced wild‐type DddA in TALEDs with DddA6 or DddA11 to create DddA6‐TALEDs and DddA11‐TALEDs with potentially enhanced editing activity (Figure 2a). DddA6 and DddA11 were both split at G1397 to make left and right halves of six TALED pairs targeting different mtDNA loci. We found that DddA11‐TALEDs were slightly less efficient than the original TALEDs (Figure 2b). By contrast, DddA6‐TALEDs with the orientation of DddA6 + L‐1397N + R‐1397C‐AD (6NC‐AD) and DddA6 + L‐1397C‐AD + R‐1397N (6CN‐AD) showed up to 18.1‐fold higher editing efficiencies than the original TALEDs at five mtDNA sites (Figure 2b).

Recently, Chen et al. reported a TadA‐8e‐derived C‐to‐G base editor (Td‐CGBE) by introducing N46L mutation in TadA‐8e.^[^ ^34^ ^]^ We generated a DddA6‐TALED variant (6CN‐AD‐N46L) using TadA‐8e‐N46L to expect C‐to‐G editing in mtDNA. However, no C‐to‐G conversion was observed for two target loci, *RNR1* (m.A1555) and *ND1* (Data not shown). In addition, TadA‐8e variants with N108Q + L145T or R111T + N127Q + Q154R mutations were found by two studies to induce significantly reduced adenine bystander editing and refine the editing window to 1–2 nucleotides.^[^ ^35^, ^36^ ^]^ Moreover, V106W substitution in TadA8e decreased both DNA and RNA off‐target activity.^[^ ^37^, ^38^ ^]^ We next evaluated the effect of these mutations in TadA8e on mtDNA editing efficiency and specificity by targeting *ND1* and *RNR1* (m.A1555) sites. Our results revealed that in contrast to V106W mutations preserving high A‐to‐G editing activity, N108Q + L145T or R111T + N127Q + Q154R mutations was not helpful for improving A‐to‐G editing efficiency or narrowing A‐to‐G editing window. Therefore, we selected the 6CN‐AD‐V106W variant with the highest mtDNA editing activity for subsequent studies (Figure S3, Supporting Information).

Similar to the strategy for optimizing DdCBE, we introduced the interface mutations (Q1310A, K1389A, T1391A, and V1411A) in DddA6 and added NES sequences in 6CN‐AD‐V106W to maintain on‐target base editing while reducing potential off‐target effect in human mtDNA and nuclear DNA (nDNA) (Figure 2c,f). We found that 6CN‐AD‐V106W containing Q1310A mutation achieved the best editing outcomes, with high A‐to‐G frequencies of 45.90−75.10% for *ND1* site and 10.01−53.24% for *RNR1* (m.A1555) site (Figure 2d,e). When 6CN‐Q1310A‐AD‐V106W fused with an additional NES1 sequence, 6CN‐Q1310A‐AD‐V106W‐NES1 substantially improved A‐to‐G editing from 41.03–71.97% to 47.39–86.65% for the *ND1* site, and also slightly elevated editing efficiency for the *RNR1* (m.A1555) site (Figure 2g,h).

We have also checked the bystander edits in the *ND1*‐TALE binding sites and sites flanking the binding sites in the presence of adenine deaminase (TadA8e). Our results demonstrated that 6CN‐Q1310A‐AD‐V106W‐NES1 variant and mitoABE groups introduced slightly higher bystander edits in the *ND1‐*TALE binding sites and sites flanking the binding sites compared to untreated group due to the presence of TadA8e (Figure S2a–c, Supporting Information). Besides, we compared the on‐target efficiencies of our high‐activity variants and the mitoBEs in mtDNA at *ND4*, *ND1*, and *ND1* (m.G3697) sites. Our results show that the two tools have their own advantages for different sites and different positions at the same site (Figure S4, Supporting Information). Therefore, rational experimental design and pre‐experimental arrangements are necessary for the selection of mitochondrial genome editing tool for manipulation of human mitochondrial DNA. Collectively, we identified the optimized version of TALEDs (6CN‐Q1310A‐AD‐V106W‐NES1) with improved A‐to‐G editing activity by combining NES sequences with the evolved DddA6 and TadA‐8e deaminases with the reported high‐fidelity mutations.

### 2.3 Characterization of Mitochondrial and Nuclear DNA Off‐Target Effects for the Engineered DdCBEs and TALEDs

**Figure 3:**
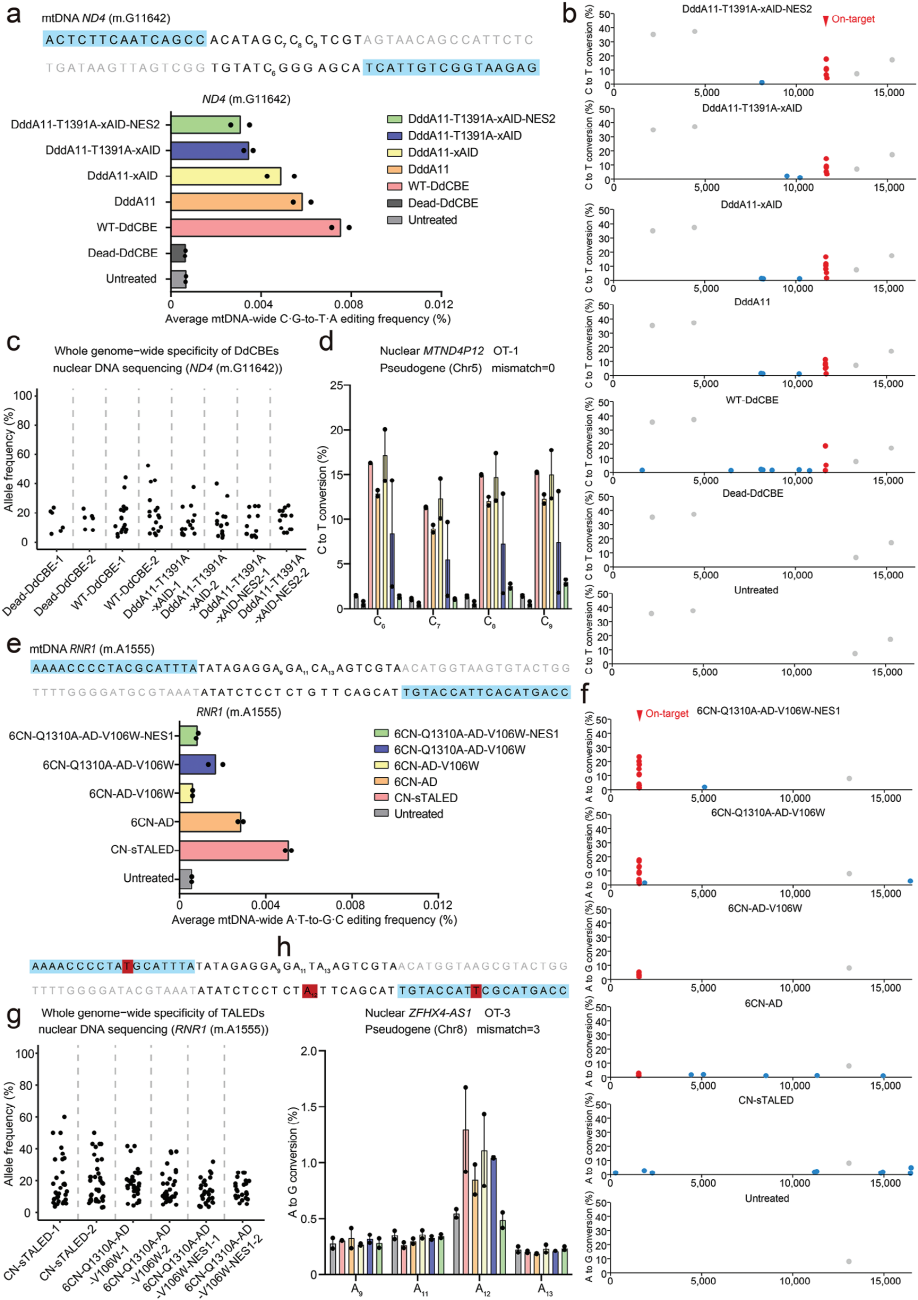
Off‐target analysis for engineered DdCBE and TALED variants targeting to the *ND4* (m.G11642) and *RNR1* (m.A1555) sites. a) The average frequencies of mitochondrial genome‐wide off‐target editing induced by Dead‐DdCBE, wild‐type DdCBE (WT‐DdCBE), DddA11, DddA11‐xAID, DddA11‐T1391A‐xAID, and DddA11‐T1391A‐xAID‐NES2 specific to the *ND4* (m.G11642) site. Error bars are s.e.m. for *n* =  2 biologically independent samples. b) Mitochondrial genome‐wide plots for C‐to‐T point mutations with frequencies ≥1%. Naturally occurring SNVs, on‐target edits (including bystander edits in the editing window) and off‐target edits are shown in gray, red, and blue, respectively. All data points from *n* = 2 biologically independent experiments are shown. c) Genome‐wide off‐target analysis for engineered DdCBE variants targeting the *ND4* (m.G11642) site. The C·G‐to‐T·A editing frequencies of each unique SNV are shown for the Dead‐DdCBE, WT‐DdCBE, and our engineered DdCBE variants. d) The corresponding nuclear DNA sequence with the greatest homology (mismatch = 0) is shown for the *ND4* (m.G11642) site. Editing efficiencies are measured by targeted deep sequencing (see Table S4 for primer sequences) (Supporting Information). Data are presented as means ± SEM. e) The average frequencies of mitochondrial genome‐wide off‐target editing induced by CN‐sTALED, 6CN‐AD, 6CN‐AD‐V106W, 6CN‐Q1310A‐AD‐V106W, and 6CN‐Q1310A‐AD‐V106W‐NES1 specific to the *RNR1* (m.A1555) site. Error bars are s.e.m. for *n* = 2 biologically independent samples. f) Mitochondrial genome‐wide plots for A‐to‐G point mutations with frequencies ≥1%. Naturally occurring SNVs, on‐target edits (including bystander edits in the editing window) and off‐target edits are shown in gray, red, and blue, respectively. All data points from *n* = 2 biologically independent experiments are shown. g) Genome‐wide off‐target analysis for engineered TALED variants targeting the *RNR1* (m.A1555) site. The A·T‐to‐G·C editing frequencies of each unique SNV are shown for CN‐sTALED and our engineered TALED variants. h) The corresponding nuclear DNA sequence with the high homology (mismatch = 3) is shown for the *RNR1* (m.A1555) site. TALE binding sites begin at N0 and are shown in blue. Nucleotide mismatches between the mtDNA and nuclear pseudogene are in red. Editing efficiencies are measured by targeted deep sequencing (see Table S4 for primer sequences) (Supporting Information). Data are presented as means ± SEM.

To analyze the off‐target activity of DdCBEs containing wild‐type DddA (WT‐DdCBE), DddA11, DddA11‐xAID, DddA11‐T1391A‐xAID, and DddA11‐T1391A‐xAID‐NES2 in the human mitochondrial and nuclear genomes, HEK293T cells were transfected with *ND4* (m.G11642)‐ and *ND1* (m.G3635)‐targeted DdCBEs for 48 h and subjected to mtDNA/nDNA‐wide whole genome sequencing and targeted deep sequencing of predicted off‐target loci in the nDNA; untreated cells or cells treated with dead DddA‐derived DdCBE (Dead‐DdCBE) served as a negative control to distinguish DdCBE‐induced single‐nucleotide variants (SNVs) from background heteroplasmy in mitochondrial genome. Our sequencing results from untreated or dead DdCBE‐treated cells revealed several naturally occurring SNVs with a heteroplasmy fraction of ≈10%, which were excluded in our analyses of mtDNA genome‐wide off‐target events. After the removal of background SNVs, the average off‐target C·G‐to‐T·A frequency for the final engineered variant of DdCBE (DddA11‐T1391A‐xAID‐NES2 with 0.0031% for *ND4* (m.G11642) site and 0.0039% for *ND1* (m.G3635) site) was lower than that of the wild‐type DdCBE (WT‐DdCBE with 0.0075% for *ND4* (m.G11642) site and 0.0055% for *ND1* (m.G3635) site) (**Figure** **3**a; Figure S5a, Supporting Information). It was found that the off‐target frequencies of these three variants (DddA11‐xAID, DddA11‐T1391‐xAID, and DddA11‐T1391‐xAID‐NES2) in the *ND4* (m.G11642) site are different with that in the *ND1* (m.G3635) site (Figure 3a; Figure S5a, Supporting information), which might be due to the different recognition sequences between *ND1* (m.G3635) and *ND4* (m.G11642) sites. In addition, the DddA11‐T1391‐xAID‐NES2 variant showed the best results in the ratio of on‐target efficiency to off‐target frequency compared to the DddA11‐xAID and DddA11‐T1391‐xAID variants (Figure S9a,b, Supporting Information). We next checked the number and position of off‐target edits induced by various DdCBE constructs in the mitochondrial genome (Figure 3b; Figure S5b, Supporting Information). The WT‐DdCBE targeting *ND4* (m.G11642) and *ND1* (m.G3635) induced off‐target C‐to‐T edits at 7 and 13 sites with conversion frequencies of ≥1.0%, respectively (Figure 3b; Figure S5b, Supporting Information). However, for other four engineered DdCBE constructs, there are only 1–3 and 3–7 off‐target edits at the *ND4* (m.G11642) and *ND1* (m.G3635) sites (Figure 3b; Figure S5b, Supporting Information). Notably, DddA11‐T1391A‐xAID‐NES2 targeting *ND4* (m.G11642) and *ND1* (m.G3635) had only one and four off‐target sites (Figure 3b; Figure S5b, Supporting Information). There is no overlapped SNVs for the majority (>70%) of off‐target sites for the five DdCBE constructs (Figure 3b; Figure S5b, Supporting Information), suggesting that DdCBE off‐target editing is largely independent of TALE‐DNA interactions.

To investigate whether our engineered DdCBE variants have TALE‐independent nuclear off‐target effects, we first performed whole genome sequencing with an average coverage of ≈30× and compared off‐target editing profile of the engineered variants (including DddA11‐T1391A‐xAID and DddA11‐T1391A‐xAID‐NES2) to that of the Dead‐DdCBE and WT‐DdCBE groups. We found that the DddA11‐T1391A‐xAID‐NES2 variant targeting *ND4* (m.G11642) group induced slightly higher level of off‐target C‐to‐T/G‐to‐A edits than Dead‐DdCBE group and fewer off‐target edits than WT‐DdCBE group (Figure 3c). Second, for the TALE‐dependent nuclear off‐target analysis of *ND4* (m.G11642)‐ and *ND1* (m.G3635)‐targeted DdCBE constructs, we examined off‐target editing of pseudogenes in nuclear genome with homology sequences as target genes in mtDNA. For two nuclear pseudogenes with 0 bp and 3 bp mismatch with *ND4* (m.G11642) target sequence, DddA11‐T1391A‐xAID‐NES2 had markedly low C‐to‐T editing rate compared to four other DdCBE constructs (Figure 3d; Figure S8a, Supporting Information). However, we did not observe >0.1% of off‐target editing at five nuclear pseudogenes for mitochondrial *ND1* (m.G3635) site, perhaps due to too many base differences from the mtDNA on‐target site (Figure S5c, Supporting Information). Finally, to investigate the off‐target effect in the presence of xAID, we characterized the ability of xAID to mediate guide‐independent off‐target DNA editing using orthogonal R‐loop assay in five dSaCas9‐induced R‐loops (Figure S6a, Supporting Information).^[^ ^39^ ^]^ Previous studies have reported that YE1‐BE3 was shown to induce no detectable off‐target effects in mouse embryos and human cell lines, while human APOBEC3A (hA3A) caused severe off‐target effects.^[^ ^39^, ^40^ ^]^ We observed the low level of off‐target editing at all five guide‐independent dSaCas9 R‐loop sites when comparing YE1‐BE3 and xAID to hA3A (Figure S6b,c, Supporting Information).

We also performed whole mitochondrial genome sequencing to evaluate off‐target profile of various TALED variants including CN‐sTALED, 6CN‐AD, 6CN‐AD‐V106W, 6CN‐Q1310A‐AD‐V106W, and 6CN‐Q1310A‐AD‐V106W‐NES1). Similar to DdCBE off‐target results, 6CN‐Q1310A‐AD‐V106W‐NES1 targeting two human mitochondrial genes (*RNR1* (m.A1555) and *ND1*) induced off‐target mutations with average frequencies of 0.0008% and 0.0034% in mtDNA, 1.8‐6.2‐fold lower than that observed with the original CN‐sTALED (Figure 3e; Figure S7a, Supporting Information). We also illustrated the ratio of on‐target editing to off‐target efficiency for each variant at *RNR1* (m.A1555) and *ND1* sites. The results implied that while 6CN‐AD‐V106W has the lowest off‐target activity, 6CN‐Q1310A‐AD‐V106W‐NES1 is the best in the ratio of on‐target editing to off‐target efficiency (Figure S9c,d, Supporting Information). In addition, the CN‐sTALED induced off‐target A‐to‐G edits at 9 and 13 sites with frequencies of ≥1.0%, whereas 6CN‐Q1310A‐AD‐V106W‐NES1 induced A‐to‐G off‐target edits at 1 and 4 sites in human mitochondrial *RNR1* (m.A1555) and *ND1* genes (Figure 3f; Figure S7b, Supporting Information). A total of 17–25 off‐targets were induced from each engineered TALEDs‐treated replicate, indicating that the majority of off‐targets were deaminase‐dependent (Figure 3f; Figure S7b, Supporting Information). Our whole genome sequencing data showed that the 6CN‐Q1310A‐AD‐V106W‐NES1 variant targeting *RNR1* (m.A1555) induced fewer off‐target A‐to‐G/T‐to‐C edits compared to the CN‐sTALED group (Figure 3g). In addition, none of these five engineered TALEDs pairs induced more than 0.5% of A‐to‐G edits at these nuclear genomic sites differing by 6–9 nucleotides from their on‐target sites in mtDNA for *ND1* site (Figure S7c, Supporting Information). For *RNR1* (m.A1555) site, we did not observe off‐target editing with the frequency of >0.4% at four nuclear pseudogenes except for the nuclear *ZFHX4‐AS1* gene at A_12_ position with ≈1% of off‐target edits, even though they differ by only 1–3 bp from the mtDNA on‐target sites (Figure 3h; Figure S8b, Supporting Information).

These data together demonstrated that our engineered DdCBE and TALED variants, especially DddA11‐T1391A‐xAID‐NES2 and 6CN‐Q1310A‐AD‐V106W‐NES1, had relatively high‐fidelity profile by inducing significantly low C‐to‐T and A‐to‐G off‐target editing in both mitochondrial and nuclear genome.

### 2.4 Installation of mtDNA Mutations with Optimized DdCBEs and TALEDs for Disease Modeling

**Figure 4:**
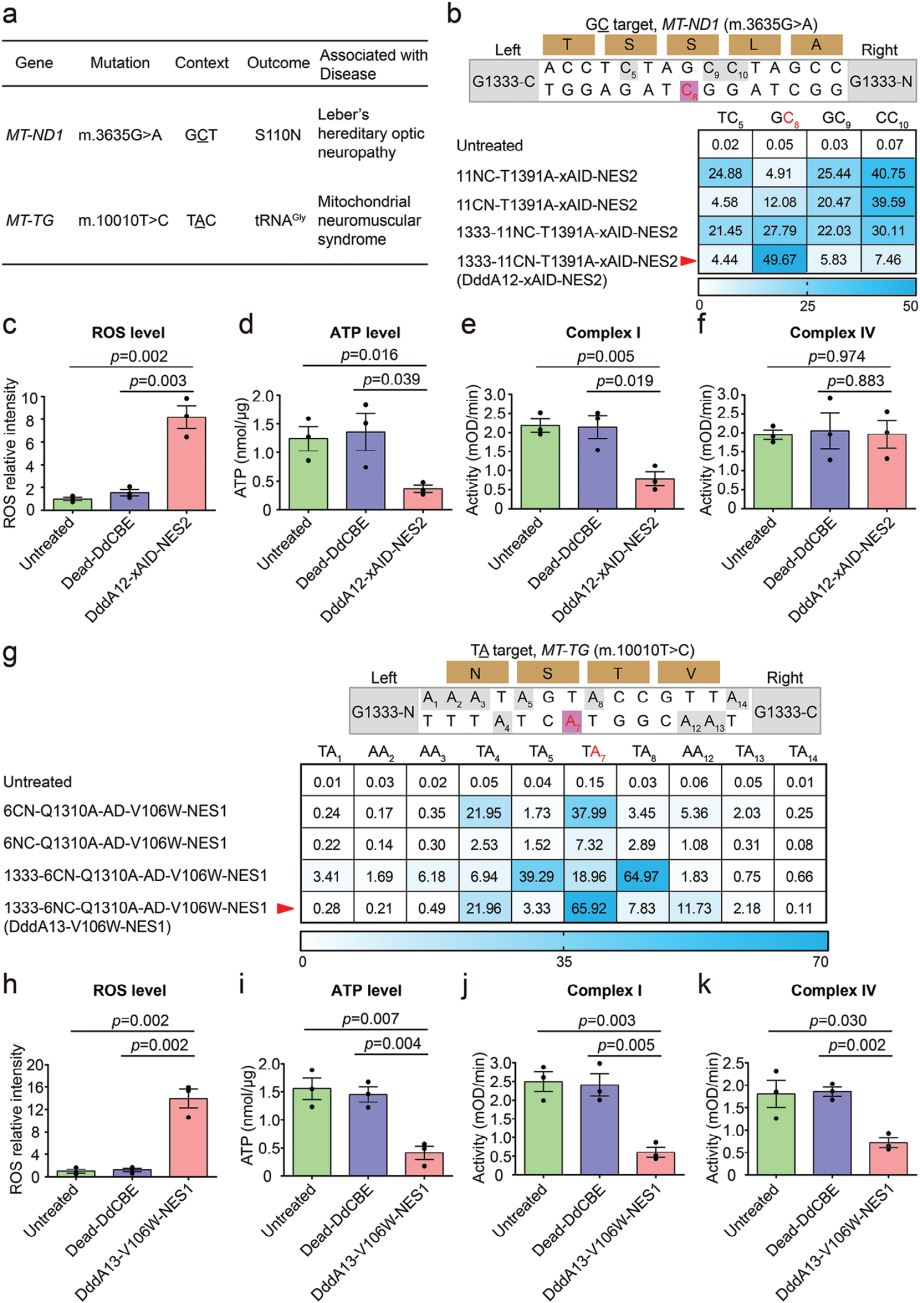
Application of high‐fidelity DdCBE and TALED variants to install pathogenic mutations in HEK293T cells. a) Using high‐fidelity DdCBE and TALED variants to install disease‐associated target mutations in human mtDNA (S, serine; N, asparagine). b) Mitochondrial C‐to‐T editing efficiencies of HEK293T cells treated with DddA11‐T1391A‐xAID‐NES2 variant at G1397 and G1333 orientation of split DddA_tox_ for the mitochondrial *ND1* (m.G3635) site in previously inaccessible GC targets. On‐target cytosines are colored red or gray, respectively. Top 30% of EGFP‐ and mCherry‐double positive cells expressing the DdCBE variants were isolated by FACS for targeted deep sequencing. The split orientation, target spacing region, and corresponding encoded amino acids are shown. 11NC‐T1391A‐xAID‐NES2, Right–G1397‐C + Left–G1397‐N orientation; 11CN‐T1391A‐xAID‐NES2, Right–G1397‐N + Left–G1397‐C orientation; 1333‐11NC‐T1391A‐xAID‐NES2, Right–G1333‐C + Left–G1333‐N orientation; 1333‐11CN‐T1391A‐xAID‐NES2 (DddA12‐xAID‐NES2), Right–G1333‐N + Left–G1333‐C orientation. Shown are means ± SEM; *n* = 3 independent experiments. The transfection time was 2 days. For the heatmap, the number is given in units of %. c–f) The levels of intracellular ROS (c), ATP (d) and the activities of complex I (e), complex IV (f) in sorted HEK293T cells treated with the DddA12‐xAID‐NES2 or Dead‐DdCBE for the *ND1* (m.G3635) site. mOD, absorbance at optical density of 450 nm (complex I activity) or 550 nm (complex IV activity). Data are presented as means ± SEM. *p* values were evaluated with the unpaired student's t‐test (two‐tailed). All data points from n  =  3 biologically independent experiments are shown. g) Mitochondrial A‐to‐G editing efficiencies of HEK293T cells treated with 6CN‐Q1310A‐AD‐V106W‐NES1 variant at G1397 and G1333 orientation of split DddA_tox_ for the mitochondrial *TG* (m.T10010) site. On‐target adenines are colored red or gray, respectively. Top 30% of EGFP‐ and mCherry‐double positive cells expressing the TALED variants were isolated by FACS for targeted deep sequencing. The split orientation, target spacing region and corresponding encoded amino acids are shown. 6CN‐Q1310A‐AD‐V106W‐NES1, Right–G1397‐N + Left–G1397‐C orientation; 6NC‐Q1310A‐AD‐V106W‐NES1, Right–G1397‐C + Left–G1397‐N orientation; 1333–6CN‐Q1310A‐AD‐V106W‐NES1, Right–G1333‐N + Left–G1333‐C orientation; 1333–6NC‐Q1310A‐AD‐V106W‐NES1 (DddA13‐V106W‐NES1), Right–G1333‐C + Left–G1333‐N orientation. Shown are means ± SEM; *n* = 3 independent experiments. The transfection time was 2 days. For the heatmap, the number is given in units of %. h–k) The levels of intracellular ROS (h), ATP (i) and the activities of complex I (j), complex IV (k) in sorted HEK293T cells treated with the DddA13‐V106W‐NES1 or Dead‐DdCBE for the *TG* (m.T10010) site. Data are presented as means ± SEM. *p* values were evaluated with the unpaired student's t‐test (two‐tailed). All data points from *n* = 3 biologically independent experiments are shown.

We demonstrated the engineered DdCBE in the present study could edit cytosine in GC context that is inaccessible for the previous DdCBE tools. To interrogate the pathogenic effect of mtDNA mutations in GC context, we designed four pairs of DdCBE based on our DddA11‐T1391A‐xAID‐NES2 variant to induce the missense m.3635G>A mutation in mitochondrial *ND1* gene. This mutation is in the GC context associated with Leber's hereditary optic neuropath (LHON) (**Figure** **4**a).^[^ ^41^, ^42^, ^43^ ^]^ We compared the editing efficiencies among DdCBEs containing DddA11‐T1391A‐xAID‐NES2 with DddA11 split at G1333 and G1397. DddA11‐T1391A‐xAID‐NES2 in Right–G1333‐N + Left–G1333‐C orientation (named DddA12‐xAID‐NES2) generated 49.67% of the edited alleles carrying desired C·G‐to‐T·A edit and a silent bystander edit with the editing rate of only 4.44% to 7.46% (Figure 4b). Unlike DddA12‐xAID‐NES2, 11NC‐T1391A‐xAID‐NES2 (Right–G1397‐C + Left–G1397‐N orientation), 11CN‐T1391A‐xAID‐NES2 (Right–G1397‐N + Left–G1397‐C orientation), and 1333‐11NC‐T1391A‐xAID‐NES2 (Right–G1333‐C + Left–G1333‐N orientation) resulted in higher bystander mutations at frequency from 4.58% to 40.75% with the average on‐target (GC_8_) efficiency of 4.91% to 27.79% (Figure 4b). We then measured the phenotypic consequences of the missense m.3635G>A mutation installed in human mtDNA by DddA12‐xAID‐NES2. We sorted for eGFP^+^mCherry^+^ cells to enrich those that expressed both halves of DddA12‐xAID‐NES2. Compared to untreated cells and cells treated with dead DdCBE, sorted cells treated with DddA12‐xAID‐NES2 exhibited increased reactive oxygen species (ROS) level and reduced ATP level (Figure 4c,d). In addition, the enzymatic activity of complex I but not of complex IV was markedly reduced in these cells (Figure 4e,f). These results revealed that DddA12‐xAID‐NES2 can install disease‐relevant mtDNA mutations in previously inaccessible GC sites with editing levels adequate to result in altered mitochondrial functions.

Lee et al. reported that their enhanced mitochondrial DNA editing tool (DdCBE‐NES + mitoTALEN) was successfully used to generate a mouse model with the m.G12918A mutation in the *ND5* gene associated with mitochondrial genetic disorders in humans.^[^ ^44^ ^]^ Consistent with the study of Lee et al., we micro‐injected DddA11‐T1391A‐xAID‐NES2 mRNA targeting *ND5* (m.G12918) into one‐cell stage mouse embryos and measured mtDNA editing frequencies in blastocysts via targeted deep sequencing. The m.G12918A mutation was obtained with frequencies of 30.3 ± 1.6% (Figure S10a,b, Supporting Information), which showed no significant difference with results of Lee et al. (36.8 ± 2.8%, DdCBE‐NES + mitoTALEN). We also assessed the toxicity of novel high‐activity variants in the mouse embryos. Developmental rates appeared unaffected for embryos injected with the high‐activity variants compared to that of PBS‐injected control embryos (Figure S10c, Supporting Information), implying no obvious toxicity for embryos treated with our high‐activity variants in the induction of efficient mitochondrial base‐editing. Therefore, based on the results of Lee et al., engineered base editors with enhanced activity and specificity or broadened sequence compatibility in our study proved to be useful for disease modeling in mice.

Next, we attempted to show the efficacy of our engineered TALEDs for the installation of disease‐relevant mutations by targeting *MT‐TG* (10010T>C) and *MT‐ATP*6 (9185T>C) that have been linked to human mitochondrial neuromuscular syndrome, Leigh syndrome, Ataxia syndrome, and NARP‐like disease (Figure 4a; Figure S11a, Supporting Information). We compared the editing efficiencies among TALEDs containing 6CN‐Q1310A‐AD‐V106W‐NES1 at that were split at G1333 and G1397. It showed that 6NC‐Q1310A‐AD‐V106W‐NES1 in Right–G1333‐C + Left–G1333‐N orientation (named DddA13‐V106W‐NES1) yielded 65.92% editing efficiency for *MT‐TG* (10010T>C) and 9.02% for *MT‐ATP*6 (9185T>C) (Figure 4g; Figure S11b, Supporting Information). In addition, we also assessed oxygen consumption rates for cells treated with *TG*‐ or *ATP6*‐targeted TALEDs. In contrast to untreated cells or control cells treated with catalytically inactive DdCBE (Dead DdCBE), cells treated with *TG*‐targeted DddA13‐V106W‐NES1 but not *ATP6*‐targeted DddA13‐V106W‐NES1 showed lower rates of ATP, and complex I and IV enzymatic activity and higher level of ROS (Figure 4h–k; Figure S11c–f, Supporting Information). The lack of phenotype by *ATP6*‐targeted DddA13‐V106W‐NES1 could be due to low mtDNA editing. Overall, our results suggested that DddA13‐V106W‐NES1 could produce mitochondrial A‐to‐G mutations with biologically significant phenotypes for disease modeling.

## 3 Discussion

In this study, we first developed DdCBEs variants by fusing different high‐fidelity mutations and ssDNA deaminases based on the evolved DddA11 variant, enabling efficient and accurate base editing within dsDNA at previously inaccessible GC targets. Compared with the recently reported DddA_tox_‐based DdCBEs^[^ ^10^ ^]^ and evolved variants (DddA6 and DddA11),^[^ ^12^ ^]^ our DddA11‐T1391A‐xAID‐NES2 improves the C‐to‐T editing efficiency and accuracy and also overcome the sequence‐context constraints, especially at GC context. Cho et al. recently found that DddA variants could function in *cis* or in *trans* with TadA8e for A‐to‐G editing in mtDNA.^[^ ^11^ ^]^ Moreover, they proposed that DddA_tox_ may transiently unwind dsDNA, exposing a few nucleotides, upon binding to dsDNA or catalyzing cytosine deamination.^[^ ^11^ ^]^ Therefore, we speculated whether DddA_tox_ can be used as a platform to make dsDNA accessible for other ssDNA‐specific cytosine deaminase with high activity to expand the scope of genome editing in mtDNA. Previous studies^[^ ^45^, ^46^ ^]^ reported that rat APOBEC1‐based base editors (named BE3) are relatively inefficient if a target C is present immediately downstream of a G. However, human APOBEC3A‐, PmCDA1, or AID‐derived base editors can mediate C‐to‐T base editing in regions with high GC dinucleotide content.^[^ ^47^, ^48^ ^]^ By screening a variety of APOBEC1, APOBEC3A, and AID deaminases, we showed that DddA11 fused with *Xenopus laevis* AID is potent at editing those GC sites (Figure 1b). At the same time, we did not detect significant TALE‐independent nuclear off‐target effects and the toxicity due to the presence of an additional ssDNA deaminase (Figure 3c,g; Figure S10c, Supporting Information). Yi et al. recently developed the mitoBEs for efficient and accurate mtDNA base editing by combining a ssDNA deaminase and nickase MutH or Nt.BspD6I(C).^[^ ^33^ ^]^ It is worth mentioning that they expanded the cytosine deaminases beyond DddA for mitochondrial C‐to‐T base editing. We therefore compared the editing efficiencies of our high‐activity variants and the mitoBEs in mtDNA at *ND4*, *ND1*, and *ND1* (m.G3697) sites. The results show that the two tools have their own advantages for different sites and different positions at the same site (Figure S4, Supporting Information). As a result, we generally recommend using different tools to manipulate the mitochondrial DNA for different situations.

Given that base editors with evolved DddA6 and DddA11 improved mtDNA editing efficiencies and expanded targeting scope,^[^ ^12^ ^]^ we develop TALEDs by replacing the wild‐type DddA_tox_ with DddA6 or DddA11 and find that DddA6‐TALEDs achieve high‐efficiency editing for five mtDNA sites from three mitochondrial genes compared to the original version. In addition, we adopt the same strategy as DdCBE engineering to optimize the specificity of DddA6‐TALEDs by introducing the reported high‐fidelity mutations of DddA and NES sequences. We show that 6CN‐Q1310A‐AD‐V106W‐NES1 variant can serve as efficient and specific tool for A‐to‐G editing in mtDNA. Overall, we believe that TALEDs with enhanced efficiency and specificity could pave the way for correction of disease‐associated mtDNA mutations in embryos, fetuses, newborns, or adult patients.

In summary, we successfully engineered DddA_tox_‐mediated DdCBEs (DddA11‐T1391A‐xAID‐NES2) and TALEDs (6CN‐Q1310A‐AD‐V106W‐NES1) that exhibited high efficiency, safety, and specificity for C‐to‐T editing at TC, CC, and GC targets and improved A‐to‐G editing in human cell lines. We also utilized the engineered variants of DdCBEs and TALEDs to install disease‐causing C‐to‐T and A‐to‐G mutations into mtDNA in human cell lines or mouse embryos. Engineered base editors with enhanced activity and specificity or broadened sequence compatibility in the study proved to be useful for mitochondrial genome study, disease modeling, and potentially therapeutic applications.

## 4 Experimental Section

### Plasmid Construction

The DdCBEs, TALEDs, and mitoBEs constructs used in this study were cloned into a mammalian expression plasmid backbone under the control of a EF1α promoter by standard molecular cloning techniques. Point mutations were introduced into DdCBE expression plasmids through site‐directed mutagenesis by PCR. Phanta Max Super‐Fidelity DNA Polymerase (Vazyme Biotech Co., Ltd) was used to amplify the insertion fragments and NES sequences, and NEBuilder HiFi DNA Assembly Master Mix (New England BioLabs) was used to perform the Gibson assembly of multiple DNA fragments. The Gibson reaction was then transformed into chemically competent *Escherichia coli* DH5α; plasmids from the transformants were subjected to Sanger sequencing to analyze the identity of the constructs. Final plasmids for mammalian cell transfection were purified using E.Z.N.A. Plasmid Mini Kit II (OMEGA) according to the manufacturer's instructions. Codon‐optimized sequences for human cell expression were obtained from GenScript. The TALE binding sequences, targeting sequences, and amino acid sequences of all DdCBEs, TALEDs, and DddA variants are provided in Table S1 and Sequences (Supporting Information).

### Mammalian Cell Culture and Transfection

HEK293T cells were cultured in DMEM (BI) supplemented with 10% fetal bovine serum (BI), 1% non‐essential amino acids (Gibco), and 1% Penicillin Streptomycin (Gibco) at 37 ^°^C with 5% CO_2_. For the DdCBEs, TALEDs, and mitoBEs efficiency assessment experiments, 8 × 10^4^ HEK293T cells were seeded onto 24‐well plates and transfected at ≈70% confluence with left half of DdCBEs, TALEDs, or mitoBEs (750 ng) and right half of DdCBEs, TALEDs, or mitoBEs (750 ng) using 3 µL Polyethylenimine (PEI) (DNA/PEI ratio of 1:2) per well. For orthogonal R‐loop assays to measure off‐target editing, 400 ng of SpCas9 guide RNA plasmid, 400 ng of SaCas9 guide RNA plasmid, 500 ng of base editor plasmid, and 500 ng of dSaCas9 plasmid were co‐transfected into HEK293T cells using 3.6 µL PEI. For controls involving no base editor, only SaCas9 guide RNA plasmid and dSaCas9 plasmid were used to maintain the total quantity of transfected DNA at 1800 ng. Top 10% of EGFP‐ and mCherry‐double positive cells were harvested from fluorescence‐activated cell sorting (FACS) 48 h after transfection. The cell lysis containing genomic and mitochondrial DNA was subjected to PCR amplification of DNA fragments including edited cytosines or adenines, and the purified PCR products were subjected to Sanger sequencing and/or targeted deep sequencing. PCR primers for mtDNA genotype analysis are indicated in Table S2 (Supporting Information). PCR primers, target protospacers, and amplicons used in orthogonal R‐loop assays are indicated in Table S5 (Supporting Information).

### Animals

Experiments involving mice were approved by the Executive Committee of Laboratory Animal Management and Ethical Review of Northwest A&F University. Super ovulated C57BL/6 females (4 weeks old) were mated with C57BL/6 males (8 weeks old). Mice were maintained in a specific pathogen‐free facility under a 12 h dark‐light cycle, and constant temperature (20–26 ^°^C) and humidity (40–60%) maintenance.

### In Vitro Transcription of DdCBE mRNA

The engineered DdCBE variant (DddA11‐T1391A‐xAID‐NES2) plasmids targeting *ND5* (m.G12918) site were constructed in the lab and protein sequences used in the study are provided in Sequences (Supporting Information). The related plasmids were linearized and used as the template for in vitro transcription using the mMESSAGE mMACHINE T7 Ultra kit (Life Technologies). The DdCBE‐variant mRNA were purified using the MEGAclear kit (Life Technologies) and eluted in RNase‐free water. In vitro transcribed RNAs were aliquoted and stored at −80 ^°^C until use. Prior to microinjection, the DdCBE‐variant mRNA was prepared by centrifuge for 10 min at 14,000 rpm at 4 ^°^C and supernatant transferred to 0.2 mL fresh PCR tubes for injection.

### Microinjection of Mouse 1‐cell Embryos with DdCBE mRNA

Super ovulated C57BL/6 females (4 weeks old) were mated with C57BL/6 males (8 weeks old), and fertilized embryos were collected from oviducts 23 h post hCG injection. For 1‐cell injection, the mixture of left TALE‐DdCBE‐variant (100 ng µL^−1^) and right TALE‐DdCBE‐variant mRNA (100 ng µL^−1^) or PBS was injected into the cytoplasm of 1‐cell embryo in a droplet of M2 medium containing 5 µg mL^−1^ cytochalasin B using a FemtoJet microinjector (Eppendorf) with constant flow settings. The injected embryos were cultured until the blastocyst stage in KSOM medium with amino acids at 37 ^°^C under 5% CO_2_ in air. Subsequently, blastocyst stage embryos were placed into PCR tubes with 2.5 µL embryo lysis buffer (0.1% Tween‐20, 0.1% Triton X‐100, 20 mg mL^−1^ proteinase K and ddH_2_O, 1:1:3:5) and incubated at 56 ^°^C for 30 min, followed by heat inactivation at 95 ^°^C for 10 min. PCR amplification was performed using nested primer sets and Phanta Max Super‐Fidelity DNA Polymerase (Vazyme Biotech Co., Ltd) and subjected to Sanger and targeted deep sequencing.

### Genomic and Mitochondrial DNA Isolation for High‐Throughput Sequencing

For the DdCBEs and TALEDs specificity optimization experiments, 1 × 10^6^ HEK293T cells were seeded onto 6‐well plates and transfected at ≈70% confluence with 2 µg of each DdCBE/TALED monomer to make up 4 µg of total plasmid DNA using PEI transfection reagent. In preparation for isolation of genomic DNA from cultured cells after transfected 48 h, the culture medium was aspirated and a lysis buffer containing proteinase K from the DNeasy Blood & Tissue Kit (Qiagen) was added to detach cells from the culture plates. Genomic DNA was extracted according to the manufacturer's instructions. Subsequently, the whole genome sequencing was performed at the mean coverage of 30× by Illumina NovaSeq 6000 platform. In addition, 1 µL of lysate was used as input for targeted deep sequencing to detect the on‐target efficiency in mtDNA and the off‐target efficiency at the predicted nuclear pseudogene loci. Primers for PCR of on‐target and off‐target regions are listed in Tables S2 and S4 (Supporting Information).

Long‐range PCR was performed on purified genomic DNA with two sets of primers (mitoWGS1‐F, mitoWGS1‐R, mitoWGS2‐F and mitoWGS2‐R in Table S3) (Supporting Information) to capture the whole mtDNA genome (two overlapping fragment of ≈8 kb each). In brief, ≈50–200 ng purified DNA was used as a template for amplification by PRIMESTAR GXL DNA polymerase (Takara). For all reactions, PRIMESTAR GXL DNA polymerase was activated at 94 ^°^C for 3 min, and PCR was performed for 35 cycles at 98 ^°^C for 30 s, 60 ^°^C for 30 s, and 72 ^°^C for 9 min, with a final extension at 72 ^°^C for 20 min. Both PCR products were purified using AMPure XP (Beckman Coulter) and subjected to tagmentation using a DNA prep kit (Illumina) following the manufacturer's protocol. Finally, the libraries were pooled and performed on the Illumina NovaSeq 6000 platform, with a sequencing depth of 1 Gb reads (≈8000× coverage) per sample.

### Analysis of Mitochondrial Genome‐Wide Off‐Target Editing

Mitochondrial genome sequencing was performed using the Illumina NovaSeq 6000 platform. To ensure data quality, Trimmomatic (v0.39) was used to trim low‐quality reads and remove adapter sequences from the FASTQ files. The resulting high‐quality reads were aligned to the GRCh38.p14 reference genome (GCF_000001405.40) with BWA‐mem (v0.7.17), and reads mapped to the mitochondrial genome were extracted from the BAM files. The processed BAM files underwent reordering, sorting, adding read groups, and marking duplicates using samtools (v1.6) and Picard (v2.3.0). Positions with conversion rates ≥ 0.1% among all bases in the mitochondrial genome were identified using the REDItoolDenovo.py script from REDItools (v.1.2.1) with parameters set as m 30 ‐q 30 ‐e ‐d ‐p ‐c 1 ‐v 1 ‐s 0. To ensure accuracy, positions with conversion rates ≥ 10% in both treated and untreated samples, representing specific SNVs in the detected cell lines, were excluded. On‐target sites for each treatment were also excluded. The remaining positions were considered as off‐target sites, and the number of edited C/G or A/T nucleotides with an editing frequency ≥0.1% was counted. The average C/G to T/A or A/T to G/C editing frequency at each base position in the off‐target sites was calculated across the mitochondrial genome. Mitochondrial genome‐wide graphs were generated by plotting the conversion rates at on‐target and off‐target sites with an editing frequency ≥ 1% across the entire mitochondrial genome. Result visualization was performed using the ggplot2 package.

### Analysis of Nuclear Genome‐Wide Off‐Target Editing

The whole genome sequencing data were initially processed using fastp (v0.22.0) for quality trimming. Following trimming, the resulting clean reads were aligned to the GRCh38 primary assembly using BWA (v0.7.17). Subsequently, samtools (v1.17) and Picard (v2.18.29) were employed for BAM file sorting and duplicate removal. To obtain potential nuclear genome off‐target editing events, this study referred to previously reported criteria to exclude the effects of high noise. Somatic variants were detected using GATK mutect2 (v4.3.0.0) in dual‐sample input mode. Only mutations meeting the following criteria were considered potential off‐target editing sites: a high median base quality (≥30), high mapping quality (≥50). Bowtie2 (v2.5.1) was employed with specific parameters (‐L 3, ‐p 4, ‐D 20, ‐R 3, and ‐a) to search for similar TALE sequences in human genome. Furthermore, Bedtools (v2.31.0) was utilized to assess whether there were overlaps between similar TALE sequences and SNVs identified by GATK.

### Measurement of Intracellular Reactive Oxygen Species (ROS)

For ROS measurement experiments, 1 × 10^6^ HEK293T cells were seeded onto 6‐well plates and transfected at ≈70% confluence with 2 µg of each DdCBE/TALED monomer to make up 4 µg of total plasmid DNA using PEI transfection reagent. Changes in the intracellular level of ROS were determined using 2′, 7′‐dichlorofluorescein diacetate (DCFH‐DA) (Beyotime, China). Culture medium was first removed and the cells were washed three times with PBS. DCFH‐DA, diluted to a final concentration of 10 µm with DMEM, was added to cultures and incubated for 20 min at 37 ^°^C. Then DCF fluorescence distribution of 1 × 10^6^ cells was monitored with excitation wavelength at 488 nm and emission wavelength at 525 nm. The increase value compared to control (untreated or Dead‐DdCBE treated cells) was viewed as the increase of intracellular ROS.

### ATP Assay

For ATP assay experiments, 1 × 10^6^ HEK293T cells were seeded onto 6‐well plates and transfected at ≈70% confluence with 2 µg of each DdCBE/TALED monomer to make up 4 µg of total plasmid DNA using PEI transfection reagent. Top 30% of EGFP‐ and mCherry‐double positive cells were harvested from FACS 48 h after transfection. The ATP content was determined by using an Enhanced ATP Assay Kit (Beyotime, China) according to the manufacturer's instructions. Briefly, after FACS, 1 × 10^6^ HEK293T cells were washed with cold PBS and lysed immediately in 200 µL lysis buffer on ice. The lysate was collected and centrifuged at 12,000 rpm for 5 min. In a 96‐well plate, 20 µL of each supernatant was added into the wells containing 100 µL ATP detection working dilution and incubated at room temperature for 5 min. The luminescence was detected by a multifunctional microplate reader (SpectraMax i3x, Molecular Devices, USA), and total ATP levels were calculated from the luminescence signals and were normalized by the cellular protein concentrations with BCA Protein Quantitative Kit (Abcam).

### Complex I and IV Activity Assay

Complex I activity assay was performed with the use of colorimetric Complex I Enzyme Activity Microplate Assay Kit (Abcam) following the manufacturer's protocol. Complex IV activity assay was performed with the use of colorimetric Complex IV Human Enzyme Activity Microplate Assay Kit (Abcam) following the manufacturer's protocol. In brief, cells were collected and washed twice with PBS (Gibco) followed by protein extraction and incubation of clarified cell lysates at a concentration of 0.25 mg mL^−1^ on the microplates for 3 h at room temperature. Complex I activity was determined by measurement of absorbance at OD = 450 nm, which was increased by reduction of a dye simultaneous to NADH to NAD^+^ oxidation. Complex IV activity was determined by measurement of absorbance at OD = 550 nm, which decreased following oxidation of reduced cytochrome c.

### Targeted Deep Sequencing

Target sites were amplified by nested PCR from genomic DNA using Phanta Max Super‐Fidelity DNA Polymerase (Vazyme Biotech Co., Ltd). The paired‐end sequencing of PCR amplicons was performed by GENEWIZ Co., Ltd using NovaSeq 6000 platform. The sequencing data were subsequently demultiplexed using fastq‐multx (v1.4.1) with the PCR primers. Sequence alignment was next performed between the demultiplexed sequencing data with each of the on‐target and off‐target sites using CRISPResso2 (v2.0.32), and then generated mapping statistics using in‐house scripts with Perl (v5.26.2) and R (v4.1.0).

### Statistical Analysis

All statistical values were presented as means ± SEM. Differences between datasets were considered to be significant at *p* value <0.05. All statistical tests were conducted with the unpaired student's *t*‐test (two‐tailed), unless otherwise stated.

## Conflict of Interest

The authors declare no conflict of interest.

## Author Contributions

Y.W., M.J., S.H., and F.Y. contributed equally to this work. Y.W., M.Z., W.L., and X.W. jointly conceived the project. Y.W., M.Z., and X.W. designed and conducted experiments. Y.W., M.J., N.R., K.X., S.L., P.G., and F.Y. assisted with plasmids construction, mouse embryo injection, FACS, PCR, and other cellular and molecular experiments. Y.W., P.G., and F.Y. performed functional experiments. S.H., Y.Z., and N.R. performed analysis of whole genome, whole mitochondrial genome, and targeted deep sequencing data. Y.W., Y.C., H.Y., W.L., M.Z., and X.W. co‐supervised the whole project. Y.W., C.X., W.L., M.Z., and X.W. wrote the manuscript.

## Supporting information

Supporting Information

## Data Availability

The data that support the findings of this study are available from the corresponding author upon reasonable request. Raw data of nuclear and mitochondrial off‐target analysis were deposited in Sequenced Read Archive (SRA): PRJNA978386.
