# Supplementary material for: Enhanced C‐To‐T and A‐To‐G Base Editing in Mitochondrial DNA with Engineered DdCBE and TALED
Source: Adv Sci (Weinh). 2023 Nov 20;11(3):2304113. doi: 10.1002/advs.202304113 (PMC10797475; doi:10.1002/advs.202304113)
Supplement: Supplementary file 1 — Supporting Information [file ADVS-11-2304113-s001.pdf]

ADVANCED SCIENCE
Open Access

Supporting Information

for *Adv. Sci.*, DOI 10.1002/advs.202304113

Enhanced C-To-T and A-To-G Base Editing in Mitochondrial DNA with Engineered DdCBE and TALED

Yinghui Wei, Ming Jin, Shuhong Huang, Fangyao Yao, Ningxin Ren, Kun Xu, Shangpu Li, Pengfei Gao, Yingsi Zhou, Yulin Chen, Hui Yang, Wen Li*, Chunlong Xu*, Meiling Zhang* and Xiaolong Wang**

Supporting Information for

Enhanced C-to-T and A-to-G base editing in mitochondrial DNA with engineered DdCBE and TALED

Yinghui Wei[1,2,#,], Ming Jin[3,#], Shuhong Huang[1,#], Fangyao Yao[1,#], Ningxin Ren[4], Kun Xu[1], Shangpu Li[1], Pengfei Gao[1], Yingsi Zhou[4], Yulin Chen[1,2], Hui Yang[4,5], Wen Li[6,*], Chunlong Xu[5,*], Meiling Zhang[6,*], Xiaolong Wang[1,2,*]*

This PDF file includes:

Figure S1 to Figure S11

Table S1 to Table S5

Supplementary sequences

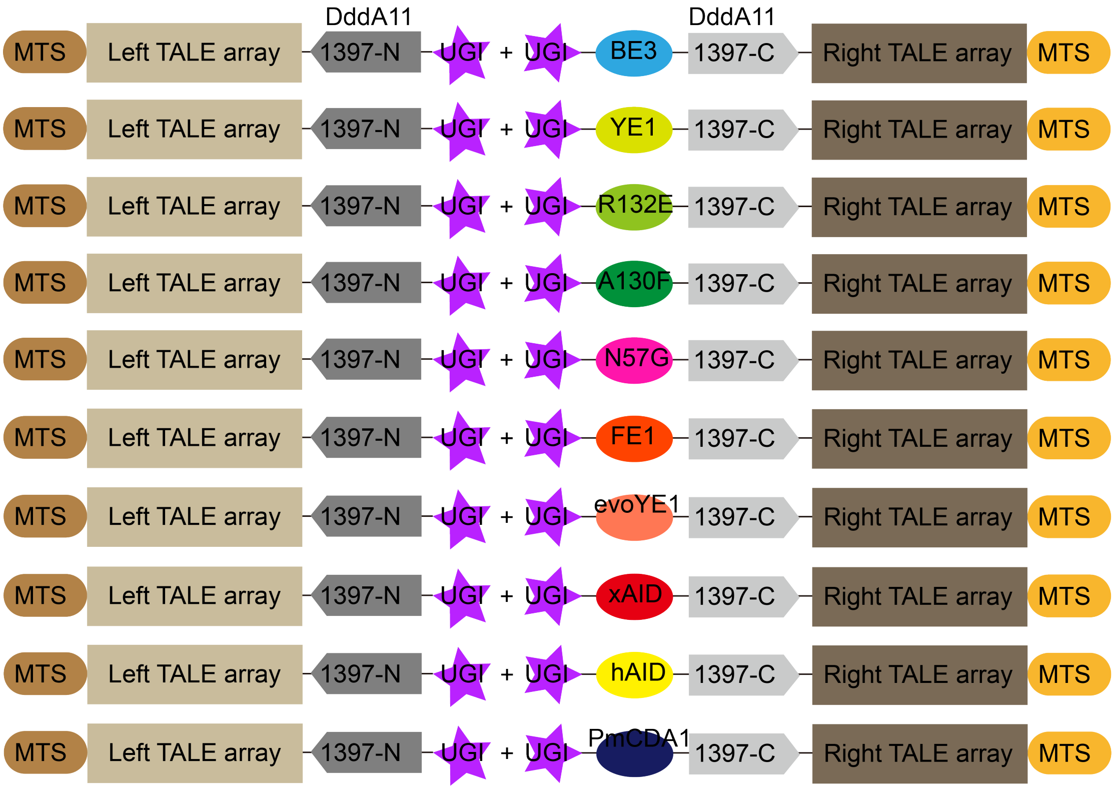

Figure S1. Schematic showing the architectures of DddA11 fused ten cytosine deaminases with ssDNA activity used in this study. MTS, mitochondrial targeting sequence; UGI, uracil glycosylase inhibitor; BE3 = rat APOBEC1; YE1 = $BE3^{W90Y+R126E}$; R132E = $BE3^{R132E}$; A130F = human $APOBE3A^{A130F}$; N57G = human $APOBE3A^{N57G}$; FE1 = $BE3^{W90F+R126E}$; evoYE1 = engineered $BE3^{W90Y+R126E}$; xAID = *Xenopus laevis* activation-induced cytidine deaminase (AID); hAID = human activation-induced cytidine deaminase (AID); PmCDA1 = *petromyzon marinus* cytidine deaminase 1.

a

mtDNA *ND4*

Left TALE — Right TALE

TGTGCTAGTAACCACGTTCT CCTGATCAAATATC ACTCTCCTACTTACAGGA

ACACGATCATTGGTGCAAGA TTACTAGTTTATAG TGAGAGGATGAATGTCCT

	G_{2}	G_{4}	C_{5}	G_{8}	C_{12}	C_{13}	C_{15}	G_{16}	C_{19}	C_{36}	C_{38}	C_{40}	C_{41}	C_{44}	C_{48}	G_{50}	G_{51}
Untreated	0.010	0.037	0.056	0.025	0.015	0.011	0.032	0.094	0.012	0.032	0.005	0.007	0.013	0.013	0.024	0.012	0.019
WT-DdCBE	0.012	0.034	0.050	0.029	0.017	0.013	0.035	0.164	0.015	0.030	0.006	0.009	0.015	0.011	0.028	0.012	0.020
11NC-T1391A-xAID-NES2	0.009	0.037	0.049	0.027	0.017	0.012	0.037	0.157	0.020	0.026	0.006	0.011	0.016	0.010	0.032	0.013	0.021
mitoCBE (right MutH)	0.009	0.042	0.054	0.028	0.070	0.047	0.107	0.154	1.119	0.051	0.018	0.042	0.015	0.012	0.024	0.012	0.031

	T_{1}	T_{3}	T_{6}	A_{7}	T_{9}	A_{10}	A_{11}	A_{14}	T_{17}	T_{18}	T_{20}	A_{35}	T_{37}	T_{39}	T_{42}	A_{43}	T_{45}	T_{46}	A_{47}	A_{49}	A_{52}
Untreated	0.020	0.018	0.021	0.032	0.012	0.047	0.035	0.056	0.018	0.022	0.026	0.084	0.024	0.033	0.022	0.016	0.024	0.021	0.015	0.037	0.021
6CN-Q1310A-AD-V106W-NES1	0.020	0.015	0.021	0.189	0.015	0.435	0.410	0.785	0.021	0.022	0.028	0.566	0.038	0.042	0.055	0.118	0.024	0.025	0.058	0.060	0.025
mitoABE (right MutH)	0.019	0.013	0.021	0.454	0.012	1.662	1.267	1.588	0.018	0.023	0.027	0.603	0.023	0.040	0.039	0.029	0.022	0.025	0.021	0.040	0.021

0 1.0 2.0 (%)

b

mtDNA *ND1*

Left TALE — Right TALE

TCTAGCCTAGCCGTTT ACTCAATCCTCTGATC AGGGTGAGCATCAAACTCAA

AGATCGGATCGGCAAA TGAGTTAGGAGACTAG TCCCACTCGTAGTTTGAGTT

	T_{1}	T_{3}	A_{4}	T_{8}	A_{9}	T_{14}	T_{15}	T_{16}	A_{33}	T_{37}	A_{39}	A_{42}	T_{43}	A_{45}	A_{46}	A_{47}	T_{49}	A_{51}	A_{52}
Untreated	0.028	0.014	0.020	0.018	0.019	0.017	0.022	0.017	0.039	0.018	0.039	0.012	0.028	0.028	0.019	0.025	0.036	0.025	0.024
6CN-Q1310A-AD-V106W-NES1	0.034	0.017	0.022	0.125	0.017	0.020	0.029	0.031	0.039	1.141	0.039	0.013	0.098	0.026	0.016	0.025	0.220	0.028	0.025
mitoABE (right MutH)	0.028	0.025	0.024	0.084	0.025	0.018	0.021	0.032	0.045	0.040	0.041	0.016	0.026	0.022	0.020	0.025	0.037	0.022	0.024

0 1.0 2.0 (%)

c

mtDNA *ND1* (m.3697)

Left TALE — Right TALE

GCATCAAACTCAAACTACGC CCTGATCGGCGCACT GCGAGCAGTAGCCCAAA

CGTAGTTTGAGTTTGATGCG GGACTAGCCGCGTGA CGCTCGTCATCGGGTTT

	A_{3}	T_{4}	A_{6}	A_{7}	A_{8}	T_{10}	A_{12}	A_{13}	A_{14}	T_{16}	A_{17}	A_{39}	A_{42}	T_{44}	A_{45}	A_{50}	A_{51}	A_{52}
Untreated	0.015	0.028	0.031	0.016	0.012	0.033	0.036	0.028	0.015	0.022	0.015	0.022	0.016	0.020	0.022	0.024	0.025	0.014
6CN-Q1310A-AD-V106W-NES1	0.140	0.029	0.117	0.035	0.068	0.046	0.173	0.043	0.104	0.311	0.576	0.016	0.053	0.099	0.043	0.030	0.023	0.025
mitoABE (right MutH)	0.128	0.028	0.161	0.026	0.058	0.059	0.336	0.059	0.142	0.070	0.362	0.025	0.039	0.019	0.026	0.023	0.025	0.016

0 1.0 2.0 (%)

Figure S2. Bystander edits in the TALE binding sites and positions flanking the binding sites. a) Bystander C-to-T and A-to-G edits in the TALE binding sites and positions flanking the binding sites for WT-DdCBE, 11NC-T1391A-xAID-NES2, mitoCBE (right MutH), 6CN-Q1310A-AD-V106W-NES1, and mitoABE (right MutH) tools at mitochondrial *ND4* site. **b-c)** Bystander A-to-G edits in the TALE binding sites and positions flanking the binding sites for CN-Q1310A-AD-V106W-NES1 and mitoABE (right MutH) tools at mitochondrial *ND1* and *ND1* (m.G3697) sites. The nucleotide adjacent to the start of the left-TALE-recognition sequence was numbered "1," and C, G, T, or A were sequentially numbered. Top 10% of EGFP- and mCherry-double positive cells were harvested from fluorescence-activated cell sorting (FACS) 48 h after transfection. The editing efficiency was tested by targeted deep sequencing. The number is given in units of %. All values are presented as the average of n = 3 biologically independent experiments.

a

Left — *RNR1* (m.A1555) — Right

G1397-C T A2 T A4 G A6 G G A9 G A11 C A13 A14 G T C G T A20 G1397-N

A1 T A3 T C T C C T C T G T T C A16 G C A19 T

	TA1	TA2	TA3	TA4	GA6	GA9	GA11	CA13	AA14	GA16	TA19	TA20
Untreated	0.01	0.01	0.02	0.06	0.05	0.08	0.13	0.04	0.05	0.02	0.01	0.01
6CN-AD-N46L	0.07	0.28	0.09	0.73	0.92	0.55	0.90	0.50	0.29	0.43	0.15	0.13
6CN-AD-V106W ►	0.24	10.98	0.81	39.79	61.72	34.77	59.30	23.07	6.99	25.54	1.99	0.28
6CN-AD-N108Q+L145T	0.08	0.31	0.09	0.78	1.13	0.60	1.01	0.48	0.33	0.51	0.15	0.14
6CN-AD-R111T+N127Q+Q154R	0.10	12.08	0.09	29.14	16.71	7.29	12.15	5.38	1.97	3.06	0.80	0.19

Color scale: 0 — 35 — 70

b

Left — *ND1* — Right

G1397-C A C T C A5 A6 T C C T C T G A T C G1397-N

T G A3 G T T A7 G G A10 G A12 C T A15 G

	GA3	CA5	AA6	GA7	GA10	CA12	GA15
Untreated	0.01	0.01	0.01	0.05	0.09	0.11	0.06
6CN-AD-N46L	0.12	0.08	0.06	0.35	0.64	0.53	0.10
6CN-AD-V106W ►	0.26	0.16	0.13	24.04	39.88	38.02	2.98
6CN-AD-N108Q+L145T	0.12	0.08	0.06	0.40	0.73	0.56	0.11
6CN-AD-R111T+N127Q+Q154R	0.13	0.08	0.08	5.83	15.67	8.63	0.39

Color scale: 0 — 20 — 40

Figure S3. Target A-to-G conversions using engineered TALED variant (6CN-AD) and recently reported several mutations of TadA8e in the mitochondrial *RNR1* (m.A1555) and *ND1* genes. a-b) Heat map showing A·T-to-G·C editing efficiencies induced by 6CN-AD with N46L, V106W, N108Q + L145T, and R111T + N127Q + Q154R mutations in HEK293T cells at two mitochondrial target sites, including *RNR1* (m.A1555) (**a**) and *ND1* (**b**). The adenines in the top strands or bottom strands are presented as A-to-G conversion frequencies. The nucleotide adjacent to the end of the left-TALE-recognition sequence was numbered "1," and A was sequentially numbered. Top 10% of EGFP- and mCherry-double positive cells were harvested from fluorescence-activated cell sorting (FACS) 48 h after transfection. The targeting efficiency was tested by targeted deep sequencing. All values are presented as the average of n = 3 biologically independent experiments. The number is given in units of %.

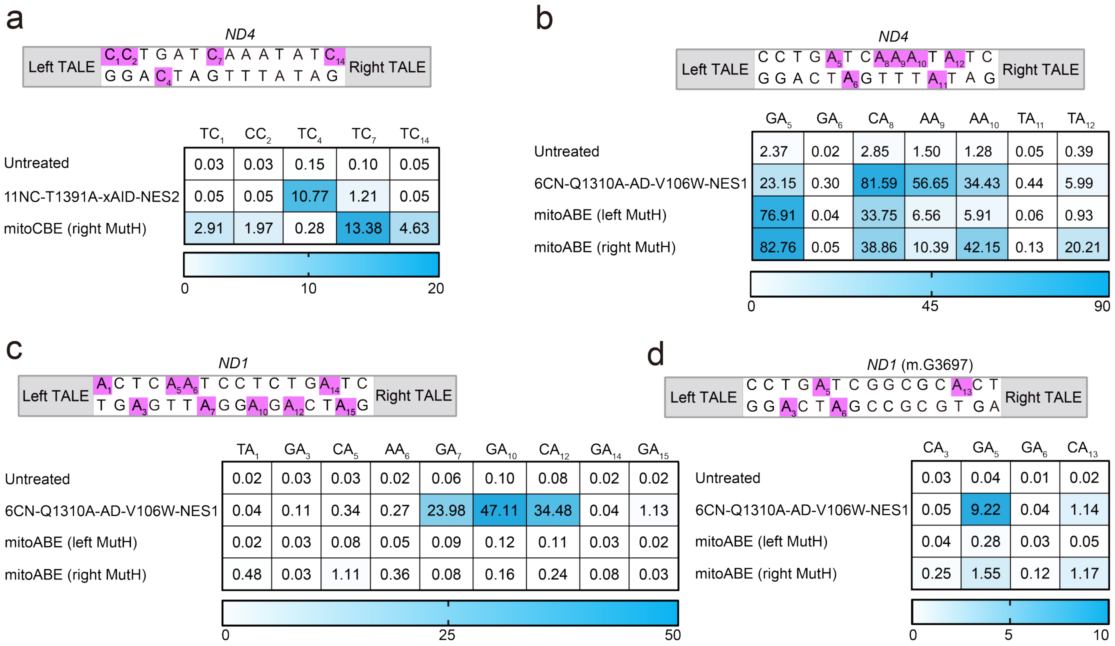

a

	TC$_1$	CC$_2$	TC$_4$	TC$_7$	TC$_{14}$
Untreated	0.03	0.03	0.15	0.10	0.05
11NC-T1391A-xAID-NES2	0.05	0.05	10.77	1.21	0.05
mitoCBE (right MutH)	2.91	1.97	0.28	13.38	4.63

b

	GA$_5$	GA$_6$	CA$_8$	AA$_9$	AA$_{10}$	TA$_{11}$	TA$_{12}$
Untreated	2.37	0.02	2.85	1.50	1.28	0.05	0.39
6CN-Q1310A-AD-V106W-NES1	23.15	0.30	81.59	56.65	34.43	0.44	5.99
mitoABE (left MutH)	76.91	0.04	33.75	6.56	5.91	0.06	0.93
mitoABE (right MutH)	82.76	0.05	38.86	10.39	42.15	0.13	20.21

c

	TA$_1$	GA$_3$	CA$_5$	AA$_6$	GA$_7$	GA$_{10}$	CA$_{12}$	GA$_{14}$	GA$_{15}$
Untreated	0.02	0.03	0.03	0.02	0.06	0.10	0.08	0.02	0.02
6CN-Q1310A-AD-V106W-NES1	0.04	0.11	0.34	0.27	23.98	47.11	34.48	0.04	1.13
mitoABE (left MutH)	0.02	0.03	0.08	0.05	0.09	0.12	0.11	0.03	0.02
mitoABE (right MutH)	0.48	0.03	1.11	0.36	0.08	0.16	0.24	0.08	0.03

d

	CA$_3$	GA$_5$	GA$_6$	CA$_{13}$
Untreated	0.03	0.04	0.01	0.02
6CN-Q1310A-AD-V106W-NES1	0.05	9.22	0.04	1.14
mitoABE (left MutH)	0.04	0.28	0.03	0.05
mitoABE (right MutH)	0.25	1.55	0.12	1.17

Figure S4. Comparison of the editing efficiency of our engineered variants with the reported mitoBEs (PMID: 37217751). a) The C-to-T editing efficiency of 11NC-T1391A-xAID-NES2 and mitoCBE targeting *ND4* in HEK293T cells. **b-d)** The A-to-G editing efficiency of 6CN-Q1310A-AD-V106W-NES1 and mitoABE targeting *ND4* (**b**), *ND1* (**c**), and *ND1* (m.G3697) (**d**) in HEK293T cells. 11NC-T1391A-xAID-NES2, Right–G1397-C + Left–G1397-N orientation; 6CN-Q1310A-AD-V106W-NES1, Right–G1397-N + Left–G1397-C orientation. All values are presented as the average of n = 3 biologically independent experiments. The number is given in units of %.

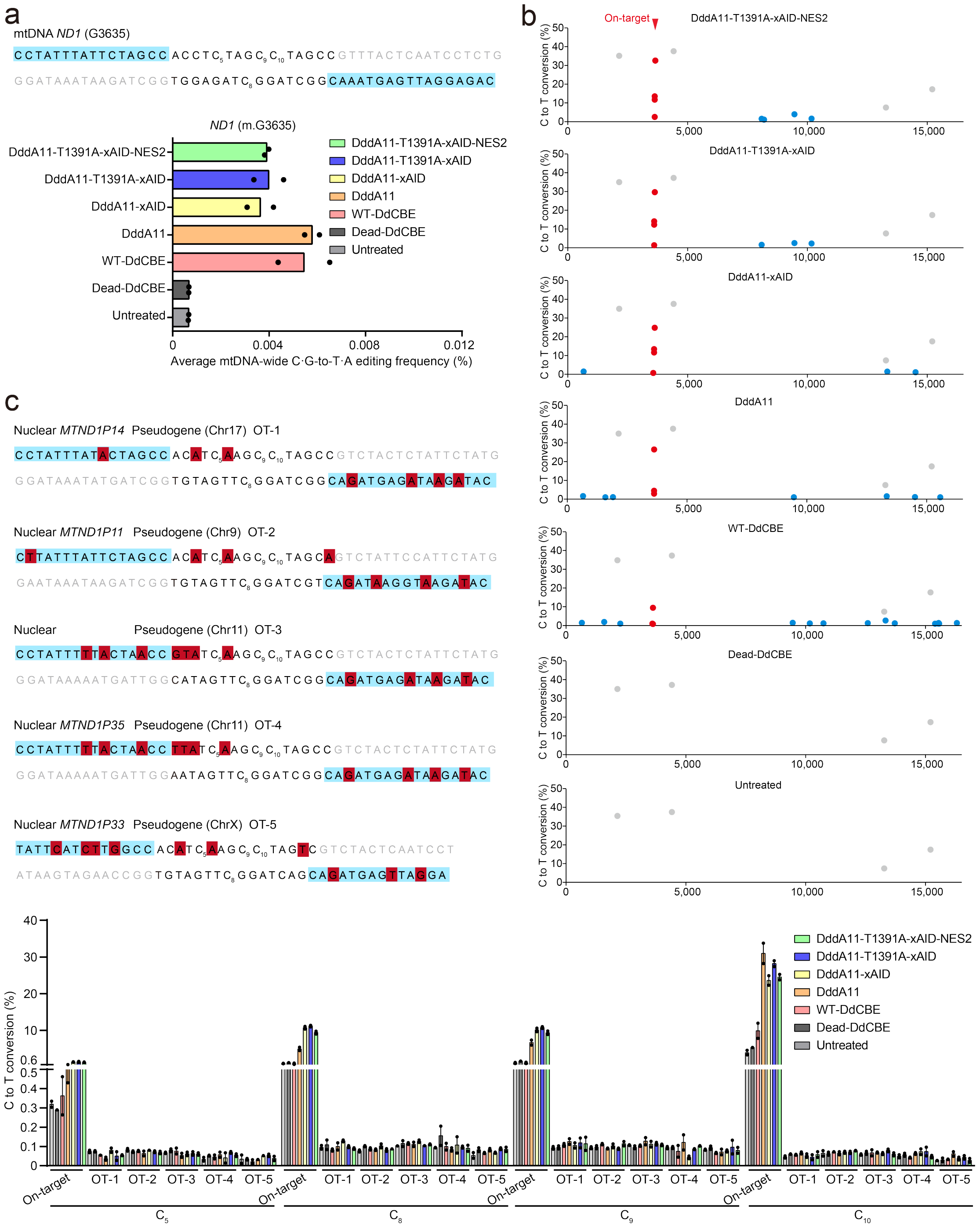

Figure S5. Off-target analysis for engineered DdCBEs specific to the *ND1* (m.G3635) site. a) The average frequencies of mitochondrial genome-wide off-target editing induced by Dead-DdCBE, wild-type DdCBE (WT-DdCBE), DddA11, DddA11-xAID, DddA11-T1391A-xAID, and DddA11-T1391A-xAID-NES2 specific to the *ND1* (m.G3635) site. Error bars are s.e.m. for n = 2 biologically independent samples. **b)** Mitochondrial genome-wide plots for C-to-T point mutations with frequencies ≥1%. Naturally occurring SNVs, on-target edits (including bystander edits in the editing window) and off-target edits are shown in blue and red, respectively. All data points from n = 2 biologically independent experiments are shown. **c)** The corresponding nuclear DNA sequences with the high homology are shown for the

ND1 (m.G3635) site. TALE binding sites begin at N0 and are shown in blue. Nucleotide mismatches between the mtDNA and nuclear pseudogene are in red. Editing efficiencies are measured by targeted deep sequencing (see Table S4 for primer sequences) (Supporting Information). Data are presented as means ± SEM.

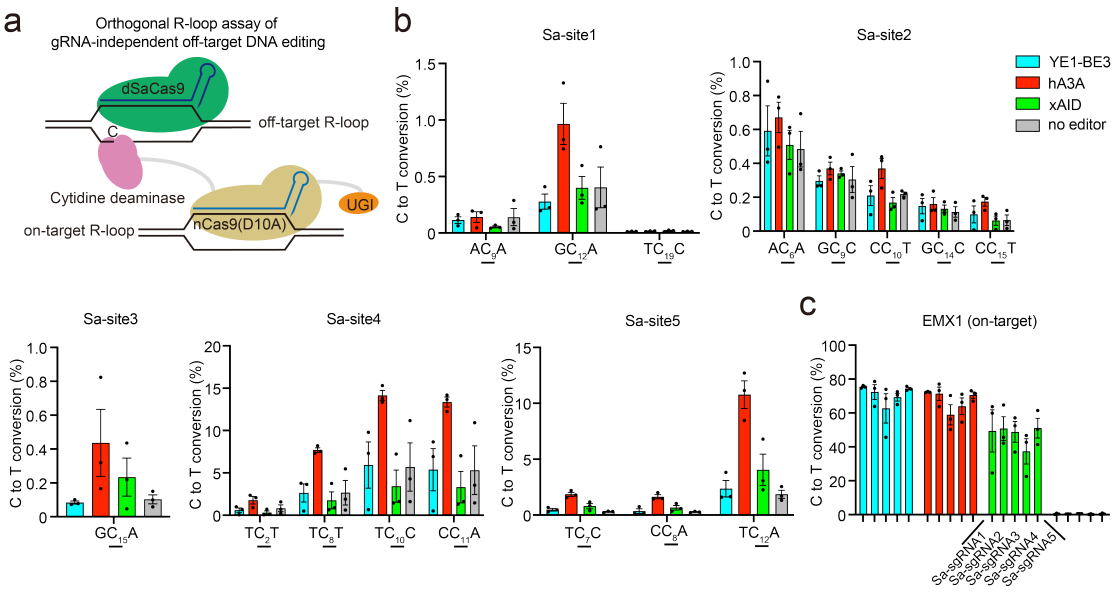

Figure S6. Sequence-independent off-target analysis for different cytosine deaminases in HEK293T cells via R-loop assay. a) Schematic of sequence-independent deamination of cytosines within dSaCas9-induced R-loop sites by deaminase fused with SpCas9. **b)** Sequence-independent off-target C•G-to-T•A editing frequencies detected by targeted high-throughput sequencing of five dSaCas9-induced R-loop loci following co-transfection with different CBEs made from fusion of SpCas9 with YE1-BE3, hA3A, and xAID. Editing efficiencies are measured by targeted deep sequencing (see Table S5 for protospacer and primer sequences) (Supporting Information). **c)** On-target DNA base editing efficiencies at the *EMX1* genomic locus corresponding to the SpCas9 sgRNA. Editing efficiencies are measured by targeted deep sequencing (see Table S5 for protospacer and primer sequences) (Supporting Information). All data points from n = 3 biologically independent experiments are shown. Data are presented as means ± SEM.

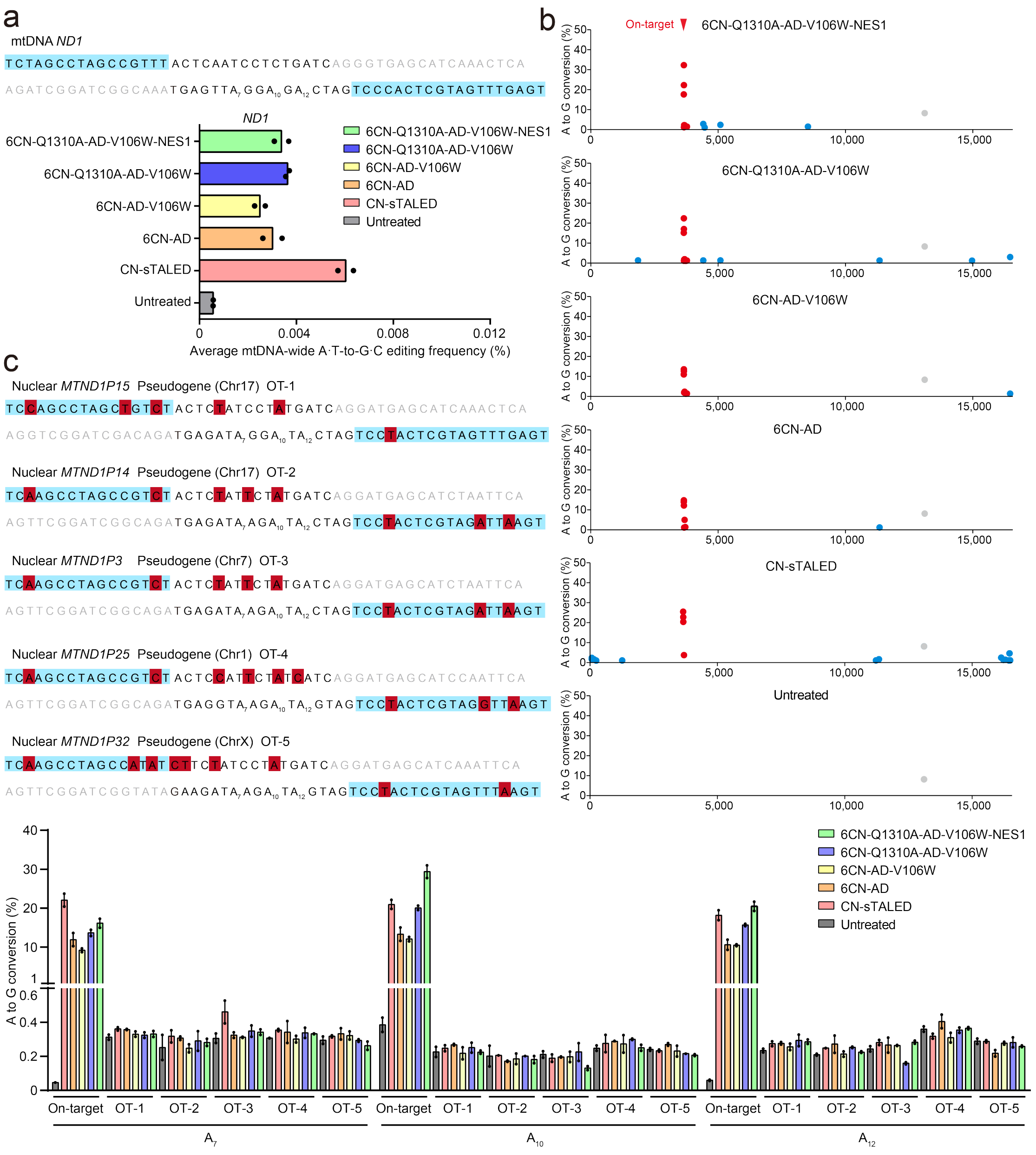

Figure S7. Off-target analysis for engineered TALEDs specific to the *ND1* site. a) The average frequencies of mitochondrial genome-wide off-target editing induced by CN-sTALED, 6CN-AD, 6CN-AD-V106W, 6CN-Q1310A-AD-V106W, and 6CN-Q1310A-AD-V106W-NES1 specific to the *ND1* site. Error bars are s.e.m. for n = 2 biologically independent samples. **b)** Mitochondrial genome-wide plots for A-to-G point mutations with frequencies ≥1%. Naturally occurring SNVs, on-target edits (including bystander edits in the editing window) and off-target edits are shown in blue and red, respectively. All data points from n = 2 biologically independent experiments are shown. **c)** The corresponding nuclear DNA sequences with the high homology are shown for the *ND1* site. TALE binding sites begin at N0 and are shown in blue. Nucleotide mismatches between the mtDNA and nuclear pseudogene are in red. Editing efficiencies are measured by targeted deep sequencing (see Table S4 for primer sequences) (Supporting Information). Data are presented as means ± SEM.

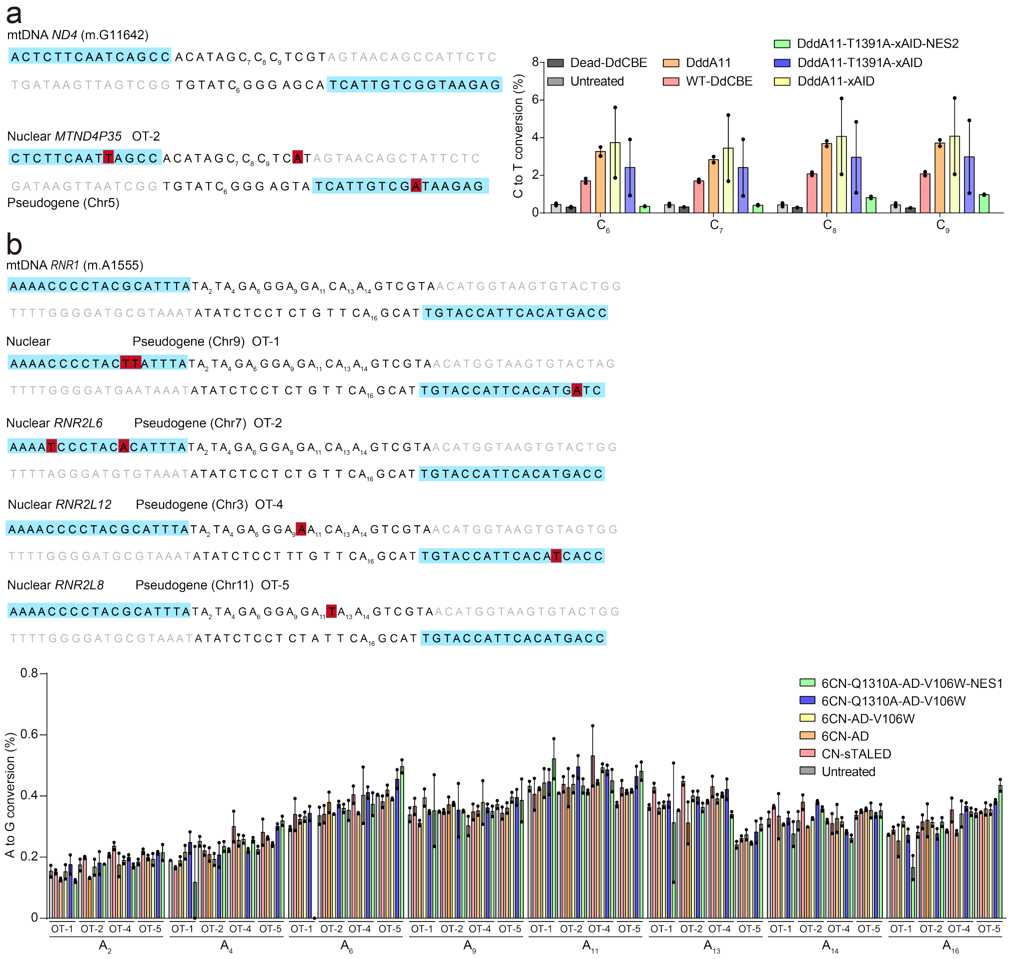

Figure S8. Off-target analysis for engineered DdCBEs and TALEDs specific to the *ND4* (m.G11642) and *RNR1* (m.A1555) sites on nuclear DNA in HEK293T cells. a) The other nuclear DNA sequence with the high homology is shown for the *ND4* (m.G11642) site. TALE binding sites begin at N0 and are shown in blue. Nucleotide mismatches between the mtDNA and nuclear pseudogene are in red. Editing efficiencies are measured by targeted deep sequencing (see Table S4 for primer sequences) (Supporting Information). Data are presented as means ± SEM. **b)** The other nuclear DNA sequence with the high homology are shown for the *RNR1* (m.A1555) site. TALE binding sites begin at N0 and are shown in blue. Nucleotide mismatches between the mtDNA and nuclear pseudogene are in red. Editing efficiencies are measured by targeted deep sequencing (see Table S4 for primer sequences) (Supporting Information). Data are presented as means ± SEM.

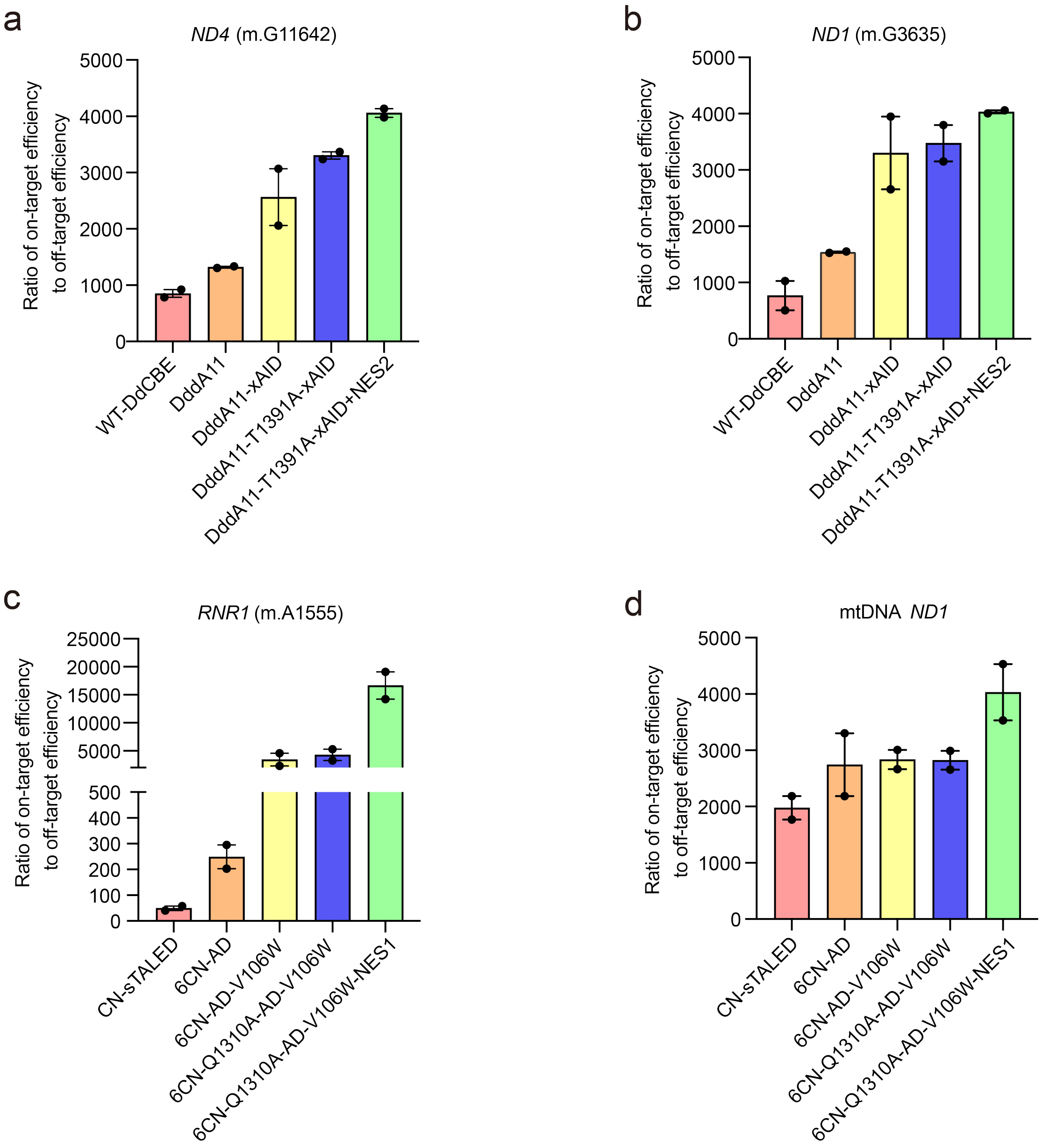

Figure S9. The ratio of on-target efficiency to off-target efficiency for each variant. a-b) The ratio of on-target efficiency to off-target efficiency for our engineered DdCBE variants at *ND4* (m.G11642) and *ND1* (m.G3635) sites. **c-d)** The ratio of on-target efficiency to off-target efficiency for our engineered TALED variants at *RNR1* (m.A1555) and *ND1* sites. All data points from n = 2 biologically independent experiments are shown. Data are presented as means ± SEM.

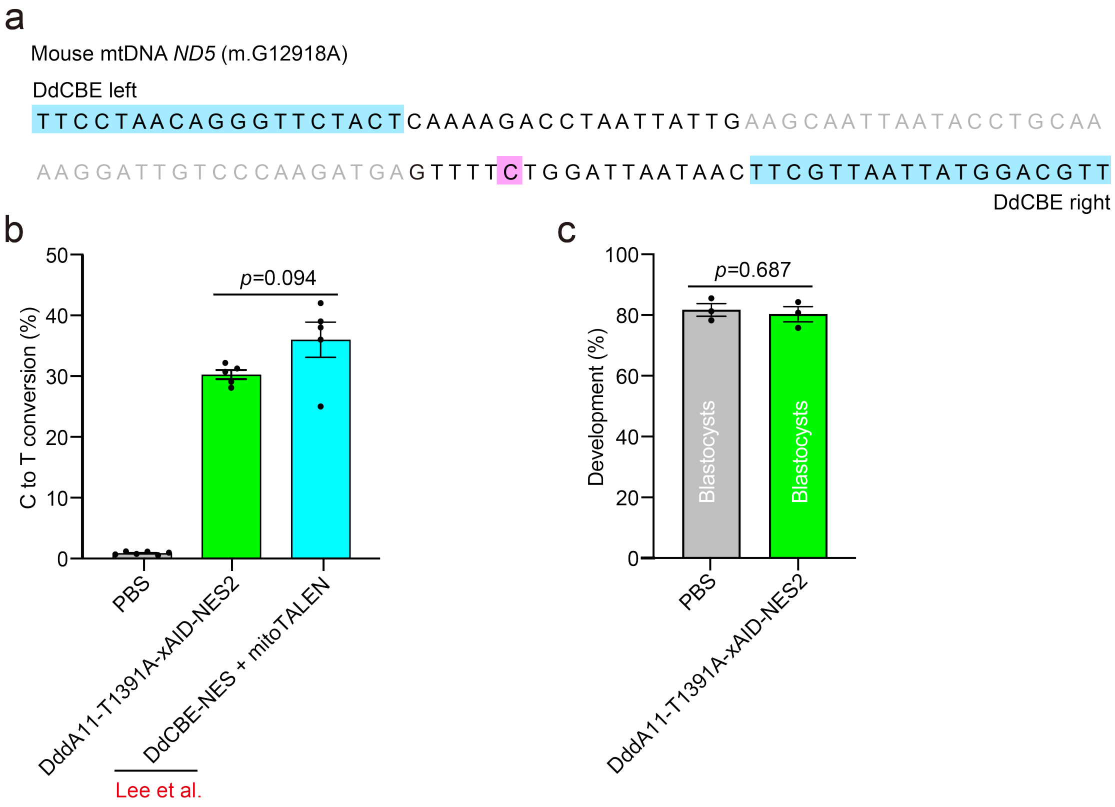

Figure S10. Engineered DdCBE variant mediates high editing efficiency in mouse blastocyst. a) Base editing target for generating the m.G12918A mutation. The TALE binding sequences for the DdCBE are highlighted in blue and for the base editing position in purple. **b)** The base editing efficiency of DddA11-T1391A-xAID-NES2 targeted *ND5* (m.G12918) in mouse blastocysts. The data of the DdCBE-NES + mitoTALEN group were cited from Lee et al.. **c)** Developmental rate for PBS-injected embryos and injected embryos with DddA11-T1391A-xAID-NES2 targeting *ND5* (m.G12918). Data are presented as the mean ± SEM. *p* values were evaluated with unpaired Student's t-test.

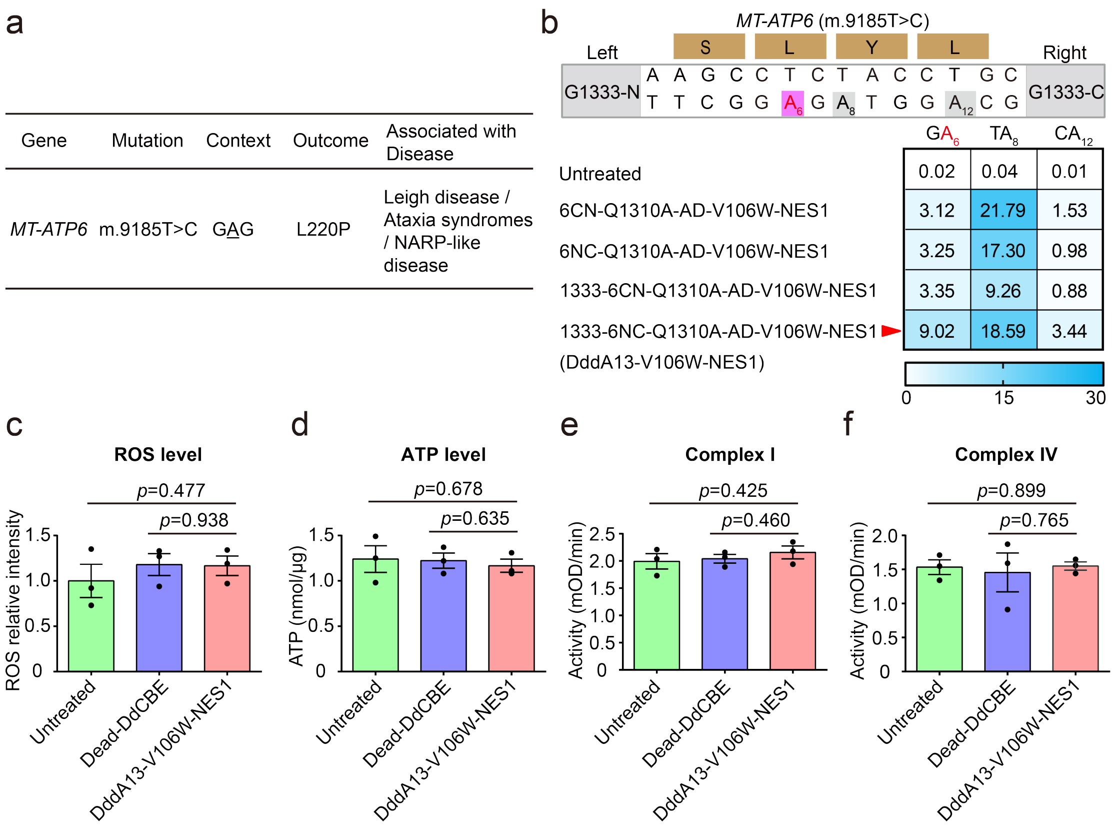

Figure S11. Application of high-fidelity TALED variant to install pathogenic mutations in HEK293T cells. a) Use high-fidelity TALED variant to install disease-associated target mutations in human mtDNA (L, leucine; P, proline). **b)** Mitochondrial A-to-G editing efficiencies of HEK293T cells treated with 6CN-Q1310A-AD-V106W-NES1 variant at G1397 and G1333 orientation of split $DddA_{tox}$ for the mitochondrial *ATP6* (m.T9185) site. On-target adenines are colored red or gray, respectively. Top 30% of EGFP- and mCherry-double positive cells expressing the TALED variants were isolated by FACS for targeted deep sequencing. The split orientation, target spacing region and corresponding encoded amino acids are shown. 6CN-Q1310A-AD-V106W-NES1, Right–G1397-N + Left–G1397-C orientation; 6NC-Q1310A-AD-V106W-NES1, Right–G1397-C + Left–G1397-N orientation; 1333-6CN-Q1310A-AD-V106W-NES1, Right–G1333-N + Left–G1333-C orientation; 1333-6NC-Q1310A-AD-V106W-NES1 (DddA13-V106W-NES1), Right–G1333-C + Left–G1333-N orientation. Shown are means ± SEM; n = 3 independent experiments. The transfection time was 2 days. **c-f)** The levels of intracellular reactive oxygen species (ROS) (**c**), ATP (**d**) and the activities of complex I (**e**), complex IV (**f**) in sorted HEK293T cells treated with the DddA13-V106W-NES1 or Dead-DdCBE for the *ATP6* (m.T9185) site. Data are presented as means ± SEM. *p* values were evaluated with the unpaired student's t-test (two-tailed). All data points from n = 3 biologically independent experiments are shown.

Table S1. TALE binding sites for DdCBEs, TALEDs, and mitoBEs.

Site	Left-mitoTALE target sequence	Right-mitoTALE target sequence
TRNL1	5’-T TTGTTAAGATG-3’	5’-T TAAGTTTTATGCGAT-3’
ND1 (m.G3635)	5’-T CCTATTTATTCTAGCC-3’	5’-T CAGAGGATTGAGTAAAC-3’
ND1 (m.G3697)	5’-T CAAACTCAAACTACGC-3’	5’-T TTGGGCTACTGCTCGC-3’
ND4 (m.G11642)	5’-T ACTCTTCAATCAGCC-3’	5’-T GAGAATGGCTGTTACT-3’
ND4	5’-T GCTAGTAACCACGTTCT-3’	5’-T CCTGTAAGTAGGAGAGT-3’
RNR1	5’-T AAAACCCCTACGCATTTA-3’	5’-T CCAGTACACTTACCATGT-3’
ND6	5’-T GACCCCCATG-3’	5’-C GATGGCTATTG-3’
ND1	5’-T CTAGCCTAGCCGTTT-3’	5’-T GAGTTTGATGCTCACCCT-3’
ATP6 (m.T9185)	5’-T TTTCACACTTCTAGT-3’	5’-T CATTATGTGTTGTCGT-3’
TRNG/TG (m.T10010)	5’-T CTTACTCTTTTAGTAT-3’	5’-T AGTTAATTGGAAGT-3’
mouse ND5 (m.G12918)	5’-T TCCTAACAGGGTTCTACT-3’	5’ -T TGCAGGTATTAATTGCTT-3’

Table S2. Primers for HTS of on-target sites targeted by DdCBEs, TALEDs, and mitoBEs.

Site	Forward primer	Reverse primer
TRNL1	GAATTCNNNNNNGGATCCCTCAACTT AGTATTATACCC	GGGTATGTTGTTAAGAAGAG
ND1 (m.G3635)	GAATTCNNNNNNGGATCCCCTGGTC AACCTCAACCTAG	TTTGGGCTACTGCTCGCAGT
ND1 (m.G3697)	ACCTCTAGCCTAGCCGTTTA	GAATTCNNNNNNGGATCCGGCTAG GGTGACTTCATATG
ND4 (m.G11642)	GAATTCNNNNNNGGATCCGCCTACG ACAAACAGACCTA	ATTATGAGAATGACTGCGCC
ND4	GAATTCNNNNNNGGATCCTAGCAAG CCTCGCTAACCTC	GGTAAATATGTAGAGGGAGT
RNR1	GAATTCNNNNNNGGATCCCCTCAAG TATACTTCAAAGG	GCTTTGTGTTAAGCTACACT
ND6	GAATTCNNNNNNGGATCCTCGCTAA CCCCACTAAAACA	TATTTAGGGGGAATGATGGT
ND1	GAATTCNNNNNNGGATCCCCTGGTC AACCTCAACCTAG	TTTGGGCTACTGCTCGCAGT
ATP6 (m.T9185)	GAATTCNNNNNNGGATCCCTATCCTA GAAATCGCTGTC	GGGTTTTACTATATGATAGG
TRNG/TG (m.T10010)	GAATTCNNNNNNGGATCCGACTATTT CTGTATGTCTCC	TAAGGCGAAGTTTATTACTC
mouse ND5 (m.G12918)	GAATTCNNNNNNGGATCCGACGAAC AAGACATCCGAAA	GAATTCNNNNNNGGATCCGCTGTT ATAGAAGTGGCGATTA

Table S3. Primers for long-range PCR of whole mitochondrial genome as two amplicons.

1st PCR	Range	1nd PCR forward (5'-3')	2nd PCR reverse (5'-3')
mitoWGS1	2478-10858 (8381bp)	GCAAATCTTACCCCGCCTG	AATTAGGCTGTGGGTGGTTG
mitoWGS2	10653-2688 (8605bp)	GCCATACTAGTCTTTGCCGC	GGCAGGTCAATTTCACTGG

Table S4. Primers for HTS of off-target sites in pseudogenes.

Site	Forward primer (5'-3')	Reverse primer (5'-3')
ND1 (m.G3635) Pseudogene (OT1)	GAATTCNNNNNNGGATCC CCCTATACCAGATCCTCTAA	GCGCCGATTAGTGCATAATT
ND1 (m.G3635) Pseudogene (OT2)	GAATTCNNNNNNGGATCC CCTATACCCCATCCCCTAAT	GTGTGCCGATCAGTACATAA
ND1 (m.G3635) Pseudogene (OT3)	GAATTCNNNNNNGGATCC CCCTATACCAGATCCTCTAA	TGTAATGCGCCAAATAGTGC
ND1 (m.G3635) Pseudogene (OT4)	GAATTCNNNNNNGGATCC CCCTATACCAGACCCTCTAA	GTAATGTGCCAATTAGTACA
ND1 (m.G3635) Pseudogene (OT5)	GAATTCNNNNNNGGATCC TCCCCATACCTTACCCCTTG	GCAATGTGCCGATCAAGGCA
ND4 (m.G11642) Pseudogene (OT1)	GAATTCNNNNNNGGATCC ACTGCGCCGGTGAAGCTTCA	CATCTGCCTGCGACAAACAG
ND4 (m.G11642) Pseudogene (OT2)	GAATTCNNNNNNGGATCC AGAATGACTGCGCCGGTGAA	TCTGCCTACGACAAACAGAC
RNR1 Pseudogene (OT1)	GAATTCNNNNNNGGATCC CACCCTCCTCAAATATTACT	GCTTTGTGTTAAGCTACACC
RNR1 Pseudogene	GAATTCNNNNNNGGATCC	CTTTGTGTTAAGCTACACCT

(OT2)	TCACCCTCCTCAAGTATCAC	
RNR1 Pseudogene (OT3)	GAATTCNNNNNNGGATCC AGCTACACCTTGGTTCGTCC	GTCAATGATTTTTGCATCAA
RNR1 Pseudogene (OT4)	GAATTCNNNNNNGGATCC AGCTACACTCTGGTTCGTCC	CCCGTCACCCTCCTCAAGTA
RNR1 Pseudogene (OT5)	GAATTCNNNNNNGGATCC GCTTTGTGTTAAGCTACACT	CGCCCGTCACCCTCCTCAAG
ND1 Pseudogene (OT1)	GAATTCNNNNNNGGATCC CTTAACTTAGGCCTTCTATT	GTTTGGGCAACTGCTCATAA
ND1 Pseudogene (OT2)	GAATTCNNNNNNGGATCC TATAGGCCTCCTATTTATAC	CCTGGGCCACAGCTCGTAAT
ND1 Pseudogene (OT3)	GAATTCNNNNNNGGATCC AGCTCGTAATGCATCGATTA	CCAAATCCCCTAATTAATTT
ND1 Pseudogene (OT4)	GAATTCNNNNNNGGATCC GGCTCGTAATATGCCGATCA	CCGGATCCCCTAATTAATTT
ND1 Pseudogene (OT5)	GAATTCNNNNNNGGATCC GCCTAAACATAGGAATGCTA	GTTTGTGCTGCTGCTCATAG

Table S5. Target protospacers and amplicons used in the orthogonal R-loop assays with corresponding primers used for genomic DNA amplification.

Site	Protospacer	PAM	Amplicon	HTS_fwd	HTS_rev
EMX1	GAGTCCGAGCAGAAGAAGAA	GGG	GAATTCNNNNNNGGATCCGTTCCAGAACCGGAGGACAAAGTACAAACGGCAGAAGCTGGAGGAGGAAGGGCCTGAGTCCGAGCAGAAGAAGAAGGGCTCCCATCACATCAACCGGTGGCGCATTGCCACGAAGCAGGCCAA	GAATTCNNNNNNGGATCCGTTCCAGAACCGGAGGACAA	TTGGCCTGCTTCGTGGCAAT
Sa-site1	GTGGTAGACAGCATGTGTCCTA	AAGGGT	GAATTCNNNNNNGGATCCTTGGGTCTACCTTACTGAGAAAATGGCCCCAGGTCATTGTCATGTCCAGTTGTGGTAGACAGCATGTGTCCTAAAGGGTATATTCACATGCATGTGCAAAAATACAGGGGTCCTTCT	GAATTCNNNNNNGGATCCTTGGGTCTACCTTACTGAGA	AGAAGGACCCCTGTATTTTT
Sa-site2	ATTTACAGCCTGGCCTTTGGGG	TCGGGT	GAATTCNNNNNNGGATCCAGAAGGGCAGGTTCCCCGAGGCGCCCAGACACCCAATCCTCCCGGTGACATTTACAGCCTGGCCTTTGGGGTCGGGTCAACGCTAGGCTGGCAGGGGAAGGGCGGGGCCGTGAGGTGA	GAATTCNNNNNNGGATCCAGAAGGGCAGGTTCCCCGAG	TCACCTCACGGCCCCGCCCTT
Sa-site3	GGTGGAGGAGGGTGCATGGGGT	CAGAAT	GAATTCNNNNNNGGATCCTCATAGAATCCTGGACAAGGTTTGAAGGACAGGTAGGATTTGGGTGGGTGGAGGAGGGTGCATGGGGTCAGAATTGTAACCGAAAACTCATTCCAGGTGGATAGAGAAAATTTCTAGTG	GAATTCNNNNNNGGATCCTCATAGAATCCTGGACAAGG	CACTAGAAATTTCTCTATCCACC
Sa-site4	TCTGCTTCTCCAGCCCTGGC	CTGGGT	GAATTCNNNNNNGGATCCGTCTATTTCTGCTGCAAGTAAGCATGCATTTGTAGGCTTGATGCTTTTTTTCTGCTTCTCCAGCCCTGGCCTGGGTCAATCCTTGGGGCCCAGACTGAGCACGTGATGGCAGAGGAAAGGAAG	GAATTCNNNNNNGGATCCGTCTATTTCTGCTGCAAGTA	CTTCCTTTCCTCTGCCATCAC
Sa-site5	GATGTTCCAATCAGTACGCA	GAGAGT	GAATTCNNNNNNGGATCCGATTGACATGCATTTCGACCAATAGCATTGCAGAGAGGCGTATCATTTCGCGGATGTTCCAATCAGTACGCAGAGAGTCGCCGTCTCCAAGGTGAAAGCGGAAGTAGGGCCTTCGCGCACC	GAATTCNNNNNNGGATCCGATTGACATGCATTTCGAC	GGTGCGCGAAGGCCCTACTT

Supplementary sequences.

Sequences used to construct DdCBE, TALED, and mitoBE architectures, and TALE amino acid sequences.

***SOD2* MTS**
LSRAVCGTSRQLAPVLGYLGSRQKHSLPD

***COX8A* MTS**
SVLTPLLLRGLTGSARRLPVPRAKIHSL

3×HA
YPYDVPDYAGYPYDVPDYAGYPYDVPDYA

3×FLAG
DYKDHDGDYKDHDIDYKDDDDK

TALE N-terminal
MDIADLRTLGYSQQQQEKIKPKVRSTVAQHHEALVGHGFTHAHIVALSQHPA
ALGTVAVKYQDMIAALPEATHEAIVGVGKQWSGARALEALLTVAGELRGPPL
QLDTGQLLKIAKRGGVTAVEAVHAWRNALTGAPLN

TALE C-terminal
SIVAQLSRPDPALAALTNDHLVALACLGGRPALDAVKKGLG

G1333 DddA$_{tox}$-N
GSYALGPYQISAPQLPAYNGQTVGTFYYVNDAGGLESKVFSSGG

G1333 DddA$_{tox}$-C
PTPYPNYANAGHVEGQSALFMRDNGISEGLVFHNNPEGTCGFCVNMTETLLP
ENAKMTVVPPEGAIPVKRGATGETKVFTGNSNSPKSPTKGGC

G1397 DddA$_{tox}$-N
GSYALGPYQISAPQLPAYNGQTVGTFYYVNDAGGLESKVFSSGGPTPYPNYA
NAGHVEGQSALFMRDNGISEGLVFHNNPEGTCGFCVNMTETLLPENAKMTV
VPPEG

G1397 DddA$_{tox}$-C
AIPVKRGATGETKVFTGNSNSPKSPTKGGC

***SOD2* 3'UTR**
ACCACGATCGTTATGCTGATCATACCCTAATGATCCCAGCAAGATAATGTCC
TGTCTTCTAAGATGTGCATCAAGCCTGGTACATACTGAAAACCCTATAAGGT
CCTGGATAATTTTTGTTTGATTATTCATTGAAGAAACATTTATTTTCCAATTG
TGTGAAGTTTTTGACTGTTAATAAAAGAATCTGTCAACCATCAAAAAAAAA
AAAAAA

***ATP5B* 3'UTR**
ACCACGATCGTTATGCTGATCATACCCTAATGATCCCAGCAAGATAATGTCC
TGTCTTCTAAGATGTGCATCAAGCCTGGTACATACTGAAAACCCTATAAGGT
CCTGGATAATTTTTGTTTGATTATTCATTGAAGAAACATTTATTTTCCAATTG

TGTGAAGTTTTTGACTGTTAATAAAAGAATCTGTCAACCATCAAAAAAAAA
AAAAAA

UGI
TNLSDIIEKETGKQLVIQESILMLPEEVEEVIGNKPESDILVHTAYDESTDENVM
LLTSDAPEYKPWALVIQDSNGENKIKML

P2A
ATNFSLLKQAGDVEENPGP

EGFP
MVSKGEELFTGVVPILVELDGDVNGHKFSVSGEGEGDATYGKLTLKFICTTGK
LPVPWPTLVTTLTYGVQCFSRYPDHMKQHDFFKSAMPEGYVQERTIFFKDDG
NYKTRAEVKFEGDTLVNRIELKGIDFKEDGNILGHKLEYNYNSHNVYIMADK
QKNGIKVNFKIRHNIEDGSVQLADHYQQNTPIGDGPVLLPDNHYLSTQSALSK
DPNEKRDHMVLLEFVTAAGITLGMDELYK

mCherry
MVSKGEEDNMAIIKEFMRFKVHMEGSVNGHEFEIEGEGEGRPYEGTQTAKLK
VTKGGPLPFAWDILSPQFMYGSKAYVKHPADIPDYLKLSFPEGFKWERVMNFE
DGGVVTVTQDSSLQDGEFIYKVKLRGTNFPSDGPVMQKKTMGWEASSERMY
PEDGALKGEIKQRLKLKDGGHYDAEVKTTYKAKKPVQLPGAYNVNIKLDITS
HNEDYTIVEQYERAEGRHSTGGMDELYK

MutH
MSQPRPLLSPPETEEQLLAQAQQLSGYTLGELAALVGLVTPENLKRDKGWIG
VLLEIWLGASAGSKPEQDFAALGVELKTIPVDSLGRPLETTFVCVAPLTGNSG
VTWETSHVRHKLKRVLWIPVEGERSIPLAQRRVGSPLLWSPNEEEDRQLRED
WEELMDMIVLGQVERITARHGEYLQIRPKAANAKALTEAIGARGERILTLPRG
FYLKKNFTSALLARHFLIQ

2aa linker
GS

3aa linker
GSG

4aa linker
SGGS

16aa linker
SGSETPGTSESATPES

The sequences of cytosine deaminase with ssDNA activity.

BE3
MSSETGPVAVDPTLRRRIEPHEFEVFFDPRELRKETCLLYEINWGGRHSIWRHT
SQNTNKHVEVNFIEKFTTERYFCPNTRCSITWFLSWSPCGECSRAITEFLSRYP

HVTLFIYIARLYHHADPRNRQGLRDLISSGVTIQIMTEQESGYCWRNFVNYSPS
NEAHWPRYPHLWVRLYVLELYCIILGLPPCLNILRRKQPQLTFFTIALQSCHYQ
RLPPHILWATGLK

YE1

MSSETGPVAVDPTLRRRIEPHEFEVFFDPRELRKETCLLYEINWGGRHSIWRHT
SQNTNKHVEVNFIEKFTTERYFCPNTRCSITWFLSYSPCGECSRAITEFLSRYPH
VTLFIYIARLYHHADPENRQGLRDLISSGVTIQIMTEQESGYCWRNFVNYSPSN
EAHWPRYPHLWVRLYVLELYCIILGLPPCLNILRRKQPQLTFFTIALQSCHYQR
LPPHILWATGLK

R132E

MSSETGPVAVDPTLRRRIEPHEFEVFFDPRELRKETCLLYEINWGGRHSIWRHT
SQNTNKHVEVNFIEKFTTERYFCPNTRCSITWFLSWSPCGECSRAITEFLSRYP
HVTLFIYIARLYHHADPRNRQGLEDLISSGVTIQIMTEQESGYCWRNFVNYSPS
NEAHWPRYPHLWVRLYVLELYCIILGLPPCLNILRRKQPQLTFFTIALQSCHYQ
RLPPHILWATGLK

A3A-130F (A130F)

MEASPASGPRHLMDPHIFTSNFNNGIGRHKTYLCYEVERLDNGTSVKMDQHR
GFLHNQAKNLLCGFYGRHAELRFLDLVPSLQLDPAQIYRVTWFISWSPCFSWG
CAGEVRAFLQENTHVRLRIFAARIFDYDPLYKEALQMLRDAGAQVSIMTYDE
FKHCWDTFVDHQGCPFQPWDGLDEHSQALSGRLRAILQNQGN

A3A-57G (N57G)

MEASPASGPRHLMDPHIFTSNFNNGIGRHKTYLCYEVERLDNGTSVKMDQHR
GFLHGQAKNLLCGFYGRHAELRFLDLVPSLQLDPAQIYRVTWFISWSPCFSWG
CAGEVRAFLQENTHVRLRIFAARIYDYDPLYKEALQMLRDAGAQVSIMTYDE
FKHCWDTFVDHQGCPFQPWDGLDEHSQALSGRLRAILQNQGN

FE1

MSSETGPVAVDPTLRRRIEPHEFEVFFDPRELRKETCLLYEINWGGRHSIWRHT
SQNTNKHVEVNFIEKFTTERYFCPNTRCSITWFLSFSPCGECSRAITEFLSRYPH
VTLFIYIARLYHHADPENRQGLRDLISSGVTIQIMTEQESGYCWRNFVNYSPSN
EAHWPRYPHLWVRLYVLELYCIILGLPPCLNILRRKQPQLTFFTIALQSCHYQR
LPPHILWATGLK

evoYE1

MSSGTGPVAVDPTLRRRIEPHEFEVFFDPRELRKETCLLYEINWGGRHSIWRHT
SQNTNKHVEVNFIEKFTTERYFCPNTRCSITWFLSYSPCGECSRAITEFLSRYPN
VTLFIYIARLYHLANPENRQGLRDLISSGVTIQIMTEQESGYCWHNFVNYSPSN
ESHWPRYPHLWVRLYVLELYCIILGLPPCLNILRRKQSQLTSFTIALQSCHYQRL
PPHILWATGLK

xAID

MTMDSMLLKRNKFIYHYKNLRWARGRHETYLCYIVKRRYSSVSCALDFGYL
RNRNGCHAEMLFLRYLSIWVGHDPHRNYRVTWFSSWSPCYDCAKRTLEFLK

GHPNFSLRIFSARLYFCEERNAEPEGLRKLQKAGVRLSVMSYKDYFYCWNTF
VETRESGFEAWDGLHENSVRLARKLRRILQPPYDMEDLREVFVLLGL

hAID
MDSLLMNRRKFLYQFKNVRWAKGRRETYLCYVVKRRDSATSFSLDFGYLRN
KNGCHVELLFLRYISDWDLDPGRCYRVTWFTSWSPCYDCARHVADFLRGNP
NLSLRIFTARLYFCEDRKAEPEGLRRLHRAGVQIAIMTFKDYFYCWNTFVENH
ERTFKAWEGLHENSVRLSRQLRRILLPLYEVDDLRDAFRTLGL

PmCDA1
MAGSTDAEYVRIHEKLDIYTFKKQFFNNKKSVSHRCYVLFELKRRGERRACF
WGYAVNKPQSGTERGIHAEIFSIRKVEEYLRDNPGQFTINWYSSWSPCADCAE
KILEWYNQELRGNGHTLKIWACKLYYEKNARNQIGLWNLRDNGVGLNVMVS
EHYQCCRKIFIQSSHNQLNENRWLEKTLKRAEKRRSELSIMIQVKILHTTKSPA
V

Nuclear export signal (NES) sequences.

NES1
LPPLERLTL

NES2
LQKKLEELELD

NES3
VDEMTKKFGTLTIHDTEK

NES4
VDEMTKKFGTLTIHDTEKGSLQLPPLERLTL

NES5
VDEMTKKFGTLTIHDTEKGSLQKKLEELELD

The sequences of TadA8e and their variants.

TadA8e (AD)
MSEVEFSHEYWMRHALTLAKRARDEREVPVGAVLVLNNRVIGEGWNRAIGL
HDPTAHAEIMALRQGGLVMQNYRLIDATLYVTFEPCVMCAGAMIHSRIGRVV
FGVRNSKRGAAGSLMNVLNYPGMNHRVEITEGILADECAALLCDFYRMPRQ
VFNAQKKAQSSIN

TadA8e-V106W (AD-V106W)
MSEVEFSHEYWMRHALTLAKRARDEREVPVGAVLVLNNRVIGEGWNRAIGL
HDPTAHAEIMALRQGGLVMQNYRLIDATLYVTFEPCVMCAGAMIHSRIGRVV
FG**W**RNSKRGAAGSLMNVLNYPGMNHRVEITEGILADECAALLCDFYRMPRQ
VFNAQKKAQSSIN

TadA8e-N46L (AD-N46L)

MSEVEFSHEYWMRHALTLAKRARDEREVPVGAVLVLNNRVIGEGW**L**RAIGL
HDPTAHAEIMALRQGGLVMQNYRLIDATLYVTFEPCVMCAGAMIHSRIGRVV
FGVRNSKRGAAGSLMNVLNYPGMNHRVEITEGILADECAALLCDFYRMPRQ
VFNAQKKAQSSIN

TadA8e-N108Q+L145T (AD-N108Q+L145T)
MSEVEFSHEYWMRHALTLAKRARDEREVPVGAVLVLNNRVIGEGWNRAIGL
HDPTAHAEIMALRQGGLVMQNYRLIDATLYVTFEPCVMCAGAMIHSRIGRVV
FGVR**Q**SKRGAAGSLMNVLNYPGMNHRVEITEGILADECAAL**T**CDFYRMPRQ
VFNAQKKAQSSIN

TadA8e-R111T+N127K+Q154R (AD-R111T+N127K+Q154R)
MSEVEFSHEYWMRHALTLAKRARDEREVPVGAVLVLNNRVIGEGWNRAIGL
HDPTAHAEIMALRQGGLVMQNYRLIDATLYVTFEPCVMCAGAMIHSRIGRVV
FGVRNSK**T**GAAGSLMNVLNYPGM**K**HRVEITEGILADECAALLCDFYRMPR**R**
VFNAQKKAQSSIN

The sequences of full-length DddA$_{tox}$ and their variants.

DddA$_{tox}$
GSYALGPYQISAPQLPAYNGQTVGTFYYVNDAGGLESKVFSSGGPTPYPNYA
NAGHVEGQSALFMRDNGISEGLVFHNNPEGTCGFCVNMTETLLPENAKMTV
VPPEGAIPVKRGATGETKVFTGNSNSPKSPTKGGC

DddA$_{tox}$-Q1310A
GSYALGPYQISAPQLPAYNG**A**TVGTFYYVNDAGGLESKVFSSGGPTPYPNYA
NAGHVEGQSALFMRDNGISEGLVFHNNPEGTCGFCVNMTETLLPENAKMTV
VPPEGAIPVKRGATGETKVFTGNSNSPKSPTKGGC

DddA$_{tox}$-K1389A
GSYALGPYQISAPQLPAYNGQTVGTFYYVNDAGGLESKVFSSGGPTPYPNYA
NAGHVEGQSALFMRDNGISEGLVFHNNPEGTCGFCVNMTETLLPENA**A**MTV
VPPEGAIPVKRGATGETKVFTGNSNSPKSPTKGGC

DddA$_{tox}$-T1391A
GSYALGPYQISAPQLPAYNGQTVGTFYYVNDAGGLESKVFSSGGPTPYPNYA
NAGHVEGQSALFMRDNGISEGLVFHNNPEGTCGFCVNMTETLLPENAKM**A**V
VPPEGAIPVKRGATGETKVFTGNSNSPKSPTKGGC

DddA$_{tox}$-V1411A
GSYALGPYQISAPQLPAYNGQTVGTFYYVNDAGGLESKVFSSGGPTPYPNYA
NAGHVEGQSALFMRDNGISEGLVFHNNPEGTCGFCVNMTETLLPENAKMTV
VPPEGAIPVKRGATGETK**A**FTGNSNSPKSPTKGGC

DddA6

GSYALGPYQISAPQLPAYNG**R**TVGTFYYVNDAGGLESKVF**I**SGGPTPYPNYA
NAGHVEGQSALFMRDNGISEGLVFHNNPEGTCGFCVNM**I**ETLLPENAKMTV
VPPEGAIPVKRGATGETKVF**I**GNSNSPKSPTKGGC

DddA11
GSYALGPYQISAPQLPAYNGQTVGTFYYVNDAGGLESKVF**I**SGGPTPYPNY**V**
SAGHVEGQSALFMRDNGISEGLVFHNNP**K**GTCGFCVNM**I**ETLLPENAKMTV
VPPEGAIPVKRGATGETKVF**I**GNSNSPKSPTKGGC

The general architecture of left-side halves of DdCBEs (from N- to C-terminus): ***SOD2* MTS - 3×HA - mitoTALE - 2aa linker - DddA$_{tox}$ half - 4aa linker - 1× UGI - (4aa linker - NES) - 3aa linker - P2A - mCheery - *SOD2* 3'UTR**

The general architecture of right-side halves of DdCBEs (from N- to C-terminus): ***COX8A* MTS - 3×FLAG - mitoTALE - 2aa linker - DddA$_{tox}$ half - 16aa linker - cytosine deaminase with ssDNA activity - 4aa linker - 1×UGI - (4aa linker - NES) - 3aa linker - P2A - EGFP - *ATP5B* 3'UTR**

or

The general architecture of left-side halves of DdCBEs (from N- to C-terminus): ***SOD2* MTS - 3×HA - mitoTALE - 2aa linker - DddA$_{tox}$ half - 16aa linker - cytosine deaminase with ssDNA activity - 4aa linker - 1×UGI - (4aa linker - NES) - 3aa linker - P2A - EGFP - *ATP5B* 3'UTR**

The general architecture of right-side halves of DdCBEs (from N- to C-terminus): ***COX8A* MTS - 3×FLAG - mitoTALE - 2aa linker - DddA$_{tox}$ half - 4aa linker - 1 ×UGI - (4aa linker - NES) - 3aa linker - P2A - mCheery - *SOD2* 3'UTR**

The general architecture of left-side halves of TALEDs (from N- to C-terminus): ***SOD2* MTS - 3×HA - mitoTALE - 2aa linker - DddA$_{tox}$ half - 16aa linker - TadA8e - (4aa linker - NES) - 3aa linker - P2A - mCheery - *SOD2* 3'UTR**

The general architecture of right-side halves of TALEDs (from N- to C-terminus): ***COX8A* MTS - 3×FLAG - mitoTALE - 2aa linker - DddA$_{tox}$ half - (4aa linker - NES) - 3aa linker - P2A - EGFP - *ATP5B* 3'UTR**

or

The general architecture of left-side halves of TALEDs (from N- to C-terminus): ***SOD2* MTS - 3×HA - mitoTALE - 2aa linker - DddA$_{tox}$ half - 3aa linker - (4aa linker - NES) - P2A - EGFP - *ATP5B* 3'UTR**

The general architecture of right-side halves of TALEDs (from N- to C-terminus): ***COX8A* MTS - 3×FLAG - mitoTALE - 2aa linker - DddA$_{tox}$ half - 16aa linker - TadA8e- (4aa linker - NES) - 3aa linker - P2A - mCheery - *SOD2* 3'UTR**

mitoCBE (right MutH)

The general architecture of left-side halves of mitoCBEs (from N- to C-terminus): ***SOD2* MTS - 3×HA - mitoTALE - 2aa linker - rAPOBEC1 - 4aa linker - 2×UGI - 3aa linker - P2A - EGFP - *ATP5B* 3'UTR**

The general architecture of right-side halves of mitoCBEs (from N- to C-terminus): ***COX8A* MTS - 3×FLAG - mitoTALE - 2aa linker - MutH - 3aa linker - P2A - mCheery - *SOD2* 3'UTR**

mitoABE (left MutH)

The general architecture of left-side halves of mitoABEs (from N- to C-terminus): ***SOD2* MTS - 3×HA - mitoTALE - 2aa linker - MutH - 3aa linker - P2A - EGFP - *ATP5B* 3'UTR**

The general architecture of right-side halves of mitoABEs (from N- to C-terminus): ***COX8A* MTS - 3×FLAG - mitoTALE - 2aa linker - TadA8e-V106W - 3aa linker - P2A - mCheery - *SOD2* 3'UTR**

mitoABE (right MutH)

The general architecture of left-side halves of mitoABEs (from N- to C-terminus): ***SOD2* MTS - 3×HA - mitoTALE - 2aa linker - TadA8e-V106W - 3aa linker - P2A - EGFP - *ATP5B* 3'UTR**

The general architecture of right-side halves of mitoABEs (from N- to C-terminus): ***COX8A* MTS - 3×FLAG - mitoTALE - 2aa linker - MutH - 3aa linker - P2A - mCheery - *SOD2* 3'UTR**

TRNL1-DdCBE Left mitoTALE repeat:

DIADLRTLGYSQQQQEKIKPKVRSTVAQHHEALVGHGFTHAHIVALSQHPAALGTVAVKYQDMIAALPEATHEAIVGVGKQWSGARALEALLTVAGELRGPPLQLDTGQLLKIAKRGGVTAVEAVHAWRNALTGAPLNLTPDQVVAIAS<u>NG</u>GGKQALETVQRLLPVLCQAHGLTPAQVVAIAS<u>NG</u>GGKQALETVQRLLPVLCQAHGLTPAQVVAIAS<u>NN</u>GGKQALETVQRLLPVLCQAHGLTPDQVVAIAS<u>NG</u>GGKQALETVQRLLPVLCQAHGLTPDQVVAIAS<u>NG</u>GGKQALETVQRLLPVLCQAHGLTPDQVVAIAS<u>NI</u>GGKQALETVQRLLPVLCQAHGLTPDQVVAIAS<u>NI</u>GGKQALETVQRLLPVLCQAHGLTPDQVVAIAS<u>NN</u>GGKQALETVQRLLPVLCQAHGLTPDQVVAIAS<u>NI</u>GGKQALETVQRLLPVLCQAHGLTPDQVVAIAS<u>NG</u>

GGKQALETVQRLLPVLCQAHGLTPDQVVAIASNNGGRPALE**SIVAQLSRPDP**
ALAALTNDHLVALACLGGRPALDAVKKGLG

TRNL1-DdCBE Right mitoTALE repeat:

DIADLRTLGYSQQQQEKIKPKVRSTVAQHHEALVGHGFTHAHIVALSQH
PAALGTVAVKYQDMIAALPEATHEAIVGVGKQWSGARALEALLTVAGE
LRGPPLQLDTGQLLKIAKRGGVTAVEAVHAWRNALTGAPLNLTPDQVVA
IASNGGGKQALETVQRLLPVLCQAHGLTPAQVVAIASNIGGKQALETVQRLL
PVLCQAHGLTPDQVVAIASNIGGKQALETVQRLLPVLCQAHGLTPDQVVAIA
SNNGGKQALETVQRLLPVLCQAHGLTPDQVVAIASNGGGKQALETVQRLLP
VLCQAHGLTPDQVVAIASNGGGKQALETVQRLLPVLCQAHGLTPAQVVAIAS
NGGGKQALETVQRLLPVLCQAHGLTPDQVVAIASNGGGKQALETVQRLLPV
LCQAHGLTPAQVVAIASNIGGKQALETVQRLLPVLCQAHGLTPDQVVAIASN
GGGKQALETVQRLLPVLCQAHGLTPAQVVAIASNNGGKQALETVQRLLPVL
CQAHGLTPDQVVAIASHDGGKQALETVQRLLPVLCQAHGLTPAQVVAIASN
NGGKQALETVQRLLPVLCQAHGLTPDQVVAIASNIGGKQALETVQRLLPVLC
QAHGLTPDQVVAIASNGGGRPALE**SIVAQLSRPDPALAALTNDHLVALACL**
GGRPALDAVKKGLG

ND1 (m.G3635)-DdCBE/TALED Left mitoTALE repeat:

DIADLRTLGYSQQQQEKIKPKVRSTVAQHHEALVGHGFTHAHIVALSQH
PAALGTVAVKYQDMIAALPEATHEAIVGVGKQWSGARALEALLTVAGE
LRGPPLQLDTGQLLKIAKRGGVTAVEAVHAWRNALTGAPLNLTPDQVVA
IASHDGGKQALETVQRLLPVLCQAHGLTPAQVVAIASHDGGKQALETVQRLL
PVLCQAHGLTPAQVVAIASNGGGKQALETVQRLLPVLCQAHGLTPAQVVAIAS
NIGGKQALETVQRLLPVLCQAHGLTPDQVVAIASNGGGKQALETVQRLLPVL
CQAHGLTPAQVVAIASNGGGKQALETVQRLLPVLCQAHGLTPDQVVAIASNG
GGKQALETVQRLLPVLCQAHGLTPAQVVAIASNIGGKQALETVQRLLPVLCQ
AHGLTPDQVVAIASNGGGKQALETVQRLLPVLCQAHGLTPAQVVAIASNGGG
KQALETVQRLLPVLCQAHGLTPDQVVAIASHDGGKQALETVQRLLPVLCQAH
GLTPAQVVAIASNGGGKQALETVQRLLPVLCQAHGLTPDQVVAIASNIGGKQA
LETVQRLLPVLCQAHGLTPDQVVAIASNNGGKQALETVQRLLPVLCQAHGLT
PDQVVAIASHDGGKQALETVQRLLPVLCQAHGLTPDQVVAIASHDGGRPALE
SIVAQLSRPDPALAALTNDHLVALACLGGRPALDAVKKGLG

ND1 (m.G3635)-DdCBE/TALED Right mitoTALE repeat:

DIADLRTLGYSQQQQEKIKPKVRSTVAQHHEALVGHGFTHAHIVALSQH
PAALGTVAVKYQDMIAALPEATHEAIVGVGKQWSGARALEALLTVAGE
LRGPPLQLDTGQLLKIAKRGGVTAVEAVHAWRNALTGAPLNLTPDQVVA
IASHDGGKQALETVQRLLPVLCQAHGLTPAQVVAIASNIGGKQALETVQRLL
PVLCQAHGLTPDQVVAIASNNGGKQALETVQRLLPVLCQAHGLTPDQVVAIA
SNIGGKQALETVQRLLPVLCQAHGLTPDQVVAIASNNGGKQALETVQRLLPV
LCQAHGLTPDQVVAIASNNGGKQALETVQRLLPVLCQAHGLTPDQVVAIASNI
GGKQALETVQRLLPVLCQAHGLTPDQVVAIASNGGGKQALETVQRLLPVLCQ

AHGLTPDQVVAIASNGGGKQALETVQRLLPVLCQAHGLTPDQVVAIASNNGG
KQALETVQRLLPVLCQAHGLTPDQVVAIASNIGGKQALETVQRLLPVLCQAH
GLTPDQVVAIASNNGGKQALETVQRLLPVLCQAHGLTPDQVVAIASNGGGKQ
ALETVQRLLPVLCQAHGLTPAQVVAIASNIGGKQALETVQRLLPVLCQAHGLT
PDQVVAIASNIGGKQALETVQRLLPVLCQAHGLTPDQVVAIASNIGGKQALET
VQRLLPVLCQAHGLTPDQVVAIASHDGGRPALE**SIVAQLSRPDPALAALTND**
HLVALACLGGRPALDAVKKGLG

ND1 (m.G3697)-DdCBE/TALED/mitoABE Left mitoTALE repeat:

DIADLRTLGYSQQQQEKIKPKVRSTVAQHHEALVGHGFTHAHIVALSQH
PAALGTVAVKYQDMIAALPEATHEAIVGVGKQWSGARALEALLTVAGE
LRGPPLQLDTGQLLKIAKRGGVTAVEAVHAWRNALTGAPLNLTPDQVVA
IASHDGGKQALETVQRLLPVLCQAHGLTPAQVVAIASNIGGKQALETVQRLL
PVLCQAHGLTPDQVVAIASNIGGKQALETVQRLLPVLCQAHGLTPDQVVAIAS
NIGGKQALETVQRLLPVLCQAHGLTPDQVVAIASHDGGKQALETVQRLLPVL
CQAHGLTPDQVVAIASNGGGKQALETVQRLLPVLCQAHGLTPDQVVAIASHD
GGKQALETVQRLLPVLCQAHGLTPDQVVAIASNIGGKQALETVQRLLPVLCQ
AHGLTPDQVVAIASNIGGKQALETVQRLLPVLCQAHGLTPDQVVAIASNIGGK
QALETVQRLLPVLCQAHGLTPDQVVAIASHDGGKQALETVQRLLPVLCQAHG
LTPDQVVAIASNGGGKQALETVQRLLPVLCQAHGLTPAQVVAIASNIGGKQAL
ETVQRLLPVLCQAHGLTPDQVVAIASHDGGKQALETVQRLLPVLCQAHGLTP
AQVVAIASNNGGKQALETVQRLLPVLCQAHGLTPDQVVAIASHDGGRPALE**SI**
VAQLSRPDPALAALTNDHLVALACLGGRPALDAVKKGLG

ND1 (m.G3697)-DdCBE/TALED/mitoABE Right mitoTALE repeat:

DIADLRTLGYSQQQQEKIKPKVRSTVAQHHEALVGHGFTHAHIVALSQH
PAALGTVAVKYQDMIAALPEATHEAIVGVGKQWSGARALEALLTVAGE
LRGPPLQLDTGQLLKIAKRGGVTAVEAVHAWRNALTGAPLNLTPDQVVA
IASNGGGKQALETVQRLLPVLCQAHGLTPAQVVAIASNGGGKQALETVQRLL
PVLCQAHGLTPAQVVAIASNNGGKQALETVQRLLPVLCQAHGLTPDQVVAIAS
NNGGKQALETVQRLLPVLCQAHGLTPDQVVAIASNNGGKQALETVQRLLPVL
CQAHGLTPDQVVAIASHDGGKQALETVQRLLPVLCQAHGLTPDQVVAIASNG
GGKQALETVQRLLPVLCQAHGLTPAQVVAIASNIGGKQALETVQRLLPVLCQ
AHGLTPDQVVAIASHDGGKQALETVQRLLPVLCQAHGLTPAQVVAIASNGGG
KQALETVQRLLPVLCQAHGLTPDQVVAIASNNGGKQALETVQRLLPVLCQAH
GLTPDQVVAIASHDGGKQALETVQRLLPVLCQAHGLTPDQVVAIASNGGGKQ
ALETVQRLLPVLCQAHGLTPAQVVAIASHDGGKQALETVQRLLPVLCQAHGL
TPDQVVAIASNNGGKQALETVQRLLPVLCQAHGLTPDQVVAIASHDGGRPAL
E**SIVAQLSRPDPALAALTNDHLVALACLGGRPALDAVKKGLG**

ND4 (m.G11642)-DdCBE Left mitoTALE repeat:

DIADLRTLGYSQQQQEKIKPKVRSTVAQHHEALVGHGFTHAHIVALSQH
PAALGTVAVKYQDMIAALPEATHEAIVGVGKQWSGARALEALLTVAGE
LRGPPLQLDTGQLLKIAKRGGVTAVEAVHAWRNALTGAPLNLTPDQVVA

IASNIGGKQALETVQRLLPVLCQAHGLTPDQVVAIASHDGGKQALETVQRLL
PVLCQAHGLTPAQVVAIASNGGGKQALETVQRLLPVLCQAHGLTPDQVVAIAS
HDGGKQALETVQRLLPVLCQAHGLTPAQVVAIASNGGGKQALETVQRLLPVL
CQAHGLTPDQVVAIASNGGGKQALETVQRLLPVLCQAHGLTPAQVVAIASHD
GGKQALETVQRLLPVLCQAHGLTPDQVVAIASNIGGKQALETVQRLLPVLCQ
AHGLTPDQVVAIASNIGGKQALETVQRLLPVLCQAHGLTPDQVVAIASNGGG
KQALETVQRLLPVLCQAHGLTPAQVVAIASHDGGKQALETVQRLLPVLCQAH
GLTPDQVVAIASNIGGKQALETVQRLLPVLCQAHGLTPDQVVAIASNNGGKQ
ALETVQRLLPVLCQAHGLTPDQVVAIASHDGGKQALETVQRLLPVLCQAHGL
TPAQVVAIASHDGGRPALE**SIVAQLSRPDPALAALTNDHLVALACLGGRPA**
LDAVKKGLG

ND4 (m.G11642)-DdCBE Right mitoTALE repeat:

DIADLRTLGYSQQQQEKIKPKVRSTVAQHHEALVGHGFTHAHIVALSQH
PAALGTVAVKYQDMIAALPEATHEAIVGVGKQWSGARALEALLTVAGE
LRGPPLQLDTGQLLKIAKRGGVTAVEAVHAWRNALTGAPLNLTPDQVVA
IASNNGGKQALETVQRLLPVLCQAHGLTPDQVVAIASNIGGKQALETVQRLL
PVLCQAHGLTPDQVVAIASNNGGKQALETVQRLLPVLCQAHGLTPDQVVAIA
SNIGGKQALETVQRLLPVLCQAHGLTPDQVVAIASNIGGKQALETVQRLLPVL
CQAHGLTPDQVVAIASNGGGKQALETVQRLLPVLCQAHGLTPDQVVAIASNN
GGKQALETVQRLLPVLCQAHGLTPDQVVAIASNNGGKQALETVQRLLPVLCQ
AHGLTPDQVVAIASHDGGKQALETVQRLLPVLCQAHGLTPAQVVAIASNGGG
KQALETVQRLLPVLCQAHGLTPDQVVAIASNNGGKQALETVQRLLPVLCQAH
GLTPDQVVAIASNGGGKQALETVQRLLPVLCQAHGLTPDQVVAIASNGGGKQ
ALETVQRLLPVLCQAHGLTPAQVVAIASNIGGKQALETVQRLLPVLCQAHGLT
PDQVVAIASHDGGKQALETVQRLLPVLCQAHGLTPAQVVAIASNGGGRPALE**S**
IVAQLSRPDPALAALTNDHLVALACLGGRPALDAVKKGLG

ND4-DdCBE/TALED/mitoBE Left mitoTALE repeat:

DIADLRTLGYSQQQQEKIKPKVRSTVAQHHEALVGHGFTHAHIVALSQH
PAALGTVAVKYQDMIAALPEATHEAIVGVGKQWSGARALEALLTVAGE
LRGPPLQLDTGQLLKIAKRGGVTAVEAVHAWRNALTGAPLNLTPEQVVA
IASNNGGKQALETVQALLPVLCQAHGLTPQQVVAIASHDGGKQALETVQRLL
PVLCQAHGLTPQQVVAIASNGGGKQALETVQRLLPVLCQAHGLTPEQVVAIA
SNIGGKQALETVQALLPVLCQAHGLTPEQVVAIASNNGGKQALETVQALLPV
LCQAHGLTPEQVVAIASNGGGKQALETVQALLPVLCQAHGLTPEQVVAIASN
IGGKQALETVQRLLPVLCQAHGLTPEQVVAIASNIGGKQALETVQALLPVLCQ
AHGLTPEQVVAIASHDGGKQALETVQRLLPVLCQAHGLTPEQVVAIASHDGG
KQALETVQALLPVLCQAHGLTPEQVVAIASNIGGKQALETVQALLPVLCQAH
GLTPEQVVAIASHDGGKQALETVQALLPVLCQAHGLTPEQVVAIASNNGGKQ
ALETVQRLLPVLCQAHGLTPQQVVAIASNGGGKQALETVQRLLPVLCQAHG
LTPEQVVAIASNGGGKQALETVQALLPVLCQAHGLTPEQVVAIASHDGGKQA
LETVQALLPVLCQAHGLTPQQVVAIASNGGGRPALE**SIVAQLSRPDPALAAL**
TNDHLVALACLGGRPALDAVKKGLG

ND4-DdCBE/TALED/mitoBE) Right mitoTALE repeat:

DIADLRTLGYSQQQQEKIKPKVRSTVAQHHEALVGHGFTHAHIVALSQH
PAALGTVAVKYQDMIAALPEATHEAIVGVGKQWSGARALEALLTVAGE
LRGPPLQLDTGQLLKIAKRGGVTAVEAVHAWRNALTGAPLNLTPEQVVA
IASHDGGKQALETVQALLPVLCQAHGLTPQQVVAIASHDGGKQALETVQRLL
PVLCQAHGLTPQQVVAIASNGGGKQALETVQRLLPVLCQAHGLTPEQVVAIA
SNNGGKQALETVQALLPVLCQAHGLTPEQVVAIASNGGGKQALETVQALLP
VLCQAHGLTPEQVVAIASNIGGKQALETVQALLPVLCQAHGLTPEQVVAIAS
NIGGKQALETVQRLLPVLCQAHGLTPEQVVAIASNNGGKQALETVQALLPVL
CQAHGLTPEQVVAIASNGGGKQALETVQRLLPVLCQAHGLTPEQVVAIASNI
GGKQALETVQRLLPVLCQAHGLTPEQVVAIASNNGGKQALETVQALLPVLC
QAHGLTPEQVVAIASNNGGKQALETVQALLPVLCQAHGLTPEQVVAIASNIG
GKQALETVQALLPVLCQAHGLTPEQVVAIASNNGGKQALETVQRLLPVLCQ
AHGLTPQQVVAIASNIGGKQALETVQRLLPVLCQAHGLTPQQVVAIASNNGG
KQALETVQRLLPVLCQAHGLTPQQVVAIASNGGGRPALE**SIVAQLSRPDPAL**
AALTNDHLVALACLGGRPALDAVKKGLG

mouse ND5 (m.G12918)-DdCBE Left mitoTALE repeat:

GIRIQDLRTLGYSQQQQEKIKPKVRSTVAQHHEALVGHGFTHAHIVALS
QHPAALGTVAVKYQDMIAALPEATHEAIVGVGKQWSGARALEALLTVA
GELRGPPLQLDTGQLLKIAKRGGVTAVEAVHAWRNALTGAPLNLTPAQV
VAIASNGGGKQALETVQRLLPVLCQAHGLTPDQVVAIASNNGGKQALETVQ
RLLPVLCQAHGLTPAQVVAIASHDGGKQALETVQRLLPVLCQAHGLTPAQV
VAIASNIGGKQALETVQRLLPVLCQAHGLTPAQVVAIASNNGGKQALETVQR
LLPVLCQDHGLTPAQVVAIASNNGGKQALETVQRLLPVLCQAHGLTPDQVV
AIASNGGGKQALETVQRLLPVLCQAHGLTPDQVVAIASNIGGKQALETVQRL
LPVLCQDHGLTPDQVVAIASNGGGKQALETVQRLLPVLCQAHGLTPEQVVAI
ASNGGGKQALETVQRLLPVLCQAHGLTPAQVVAIASNIGGKQALETVQRLLP
VLCQDHGLTPAQVVAIASNIGGKQALETVQRLLPVLCQAHGLTPEQVVAIAS
NGGGKQALETVQRLLPVLCQAHGLTPAQVVAIASNGGGKQALETVQRLLPV
LCQAHGLTPDQVVAIASNNGGKQALETVQRLLPVLCQDHGLTPDQVVAIASH
DGGKQALETVQRLLPVLCQAHGLTPAQVVAIASNGGGKQALETVQRLLPVL
CQDHGLTPEQVVAIASNGGGKQALE**SIVAQLSRPDPALAALTNDHLVALAC**
LGGRPALDAVKKGLGGS

mouse ND5 (m.G12918)-DdCBE Right mitoTALE repeat:

GIRIQDLRTLGYSQQQQEKIKPKVRSTVAQHHEALVGHGFTHAHIVALS
QHPAALGTVAVKYQDMIAALPEATHEAIVGVGKQWSGARALEALLTVA
GELRGPPLQLDTGQLLKIAKRGGVTAVEAVHAWRNALTGAPLNLTPAQV
VAIASNGGGKQALETVQRLLPVLCQDHGLTPAQVVAIASHDGGKQALETVQ
RLLPVLCQAHGLTPEQVVAIASHDGGKQALETVQRLLPVLCQAHGLTPEQVV
AIASNGGGKQALETVQRLLPVLCQAHGLTPAQVVAIASNIGGKQALETVQRL
LPVLCQDHGLTPAQVVAIASNIGGKQALETVQRLLPVLCQAHGLTPEQVVAI
ASHDGGKQALETVQRLLPVLCQAHGLTPAQVVAIASNIGGKQALETVQRLLP

VLCQAHGLTPAQVVAIASNNGGKQALETVQRLLPVLCQAHGLTPAQVVAIAS
NNGGKQALETVQRLLPVLCQDHGLTPAQVVAIASNNGGKQALETVQRLLPV
LCQDHGLTPDQVVAIASNGGGKQALETVQRLLPVLCQAHGLTPEQVVAIASN
GGGKQALETVQRLLPVLCQAHGLTPDQVVAIASHDGGKQALETVQRLLPVL
CQDHGLTPDQVVAIASNGGGKQALETVQRLLPVLCQAHGLTPEQVVAIASNI
GGKQALETVQRLLPVLCQAHGLTPAQVVAIASHDGGKQALETVQRLLPVLC
QDHGLTPEQVVAIASNGGGKQALE**SIVAQLSRPDPALAALTNDHLVALACL**
GGRPALDAVKKGLGGS

RNR1-DdCBE/TALED Left mitoTALE repeat:

DIADLRTLGYSQQQQEKIKPKVRSTVAQHHEALVGHGFTHAHIVALSQH
PAALGTVAVKYQDMIAALPEATHEAIVGVGKQWSGARALEALLTVAGE
LRGPPLQLDTGQLLKIAKRGGVTAVEAVHAWRNALTGAPLNLTPAQVVAI
ASNIGGKQALETVQRLLPVLCQAHGLTPDQVVAIASNIGGKQALETVQRLL
PVLCQAHGLTPDQVVAIASNIGGKQALETVQRLLPVLCQAHGLTPDQVVAIAS
NIGGKQALETVQRLLPVLCQAHGLTPDQVVAIASHDGGKQALETVQRLLPVL
CQAHGLTPAQVVAIASHDGGKQALETVQRLLPVLCQAHGLTPDQVVAIASHD
GGKQALETVQRLLPVLCQAHGLTPAQVVAIASHDGGKQALETVQRLLPVLCQ
AHGLTPDQVVAIASNGGGKQALETVQRLLPVLCQAHGLTPAQVVAIASNIGGK
QALETVQRLLPVLCQAHGLTPDQVVAIASHDGGKQALETVQRLLPVLCQAHG
LTPAQVVAIASNNGGKQALETVQRLLPVLCQAHGLTPDQVVAIASHDGGKQA
LETVQRLLPVLCQAHGLTPAQVVAIASNIGGKQALETVQRLLPVLCQAHGLTP
DQVVAIASNGGGKQALETVQRLLPVLCQAHGLTPAQVVAIASNGGGKQALET
VQRLLPVLCQAHGLTPDQVVAIASNGGGKQALETVQRLLPVLCQAHGLTPAQ
VVAIASNIGGRPALE**SIVAQLSRPDPALAALTNDHLVALACLGGRPALDAV**
KKGLG

RNR1-DdCBE/TALED Right mitoTALE repeat:

DIADLRTLGYSQQQQEKIKPKVRSTVAQHHEALVGHGFTHAHIVALSQH
PAALGTVAVKYQDMIAALPEATHEAIVGVGKQWSGARALEALLTVAGE
LRGPPLQLDTGQLLKIAKRGGVTAVEAVHAWRNALTGAPLNLTPDQVVA
IASHDGGKQALETVQRLLPVLCQAHGLTPAQVVAIASHDGGKQALETVQRLL
PVLCQAHGLTPAQVVAIASNIGGKQALETVQRLLPVLCQAHGLTPDQVVAIAS
NNGGKQALETVQRLLPVLCQAHGLTPDQVVAIASNGGGKQALETVQRLLPVL
CQAHGLTPAQVVAIASNIGGKQALETVQRLLPVLCQAHGLTPDQVVAIASHDG
GKQALETVQRLLPVLCQAHGLTPAQVVAIASNIGGKQALETVQRLLPVLCQA
HGLTPDQVVAIASHDGGKQALETVQRLLPVLCQAHGLTPAQVVAIASNGGGK
QALETVQRLLPVLCQAHGLTPDQVVAIASNGGGKQALETVQRLLPVLCQAHG
LTPAQVVAIASNIGGKQALETVQRLLPVLCQAHGLTPDQVVAIASHDGGKQAL
ETVQRLLPVLCQAHGLTPAQVVAIASHDGGKQALETVQRLLPVLCQAHGLTP
DQVVAIASNIGGKQALETVQRLLPVLCQAHGLTPDQVVAIASNGGGKQALET
VQRLLPVLCQAHGLTPDQVVAIASNNGGKQALETVQRLLPVLCQAHGLTPDQ
VVAIASNGGGRPALE**SIVAQLSRPDPALAALTNDHLVALACLGGRPALDAV**
KKGLG

ND6-TALED Left mitoTALE repeat:

DIADLRTLGYSQQQQEKIKPKVRSTVAQHHEALVGHGFTHAHIVALSQH
PAALGTVAVKYQDMIAALPEATHEAIVGVGKQWSGARALEALLTVAGE
LRGPPLQLDTGQLLKIAKRGGVTAVEAVHAWRNALTGAPLNLTPQQVVA
IASNNGGKQALETVQRLLPVLCQAHGLTPEQVVAIASNIGGKQALETVQALL
PVLCQAHGLTPEQVVAIASHDGGKQALETVQRLLPVLCQAHGLTPEQVVAIA
SHDGGKQALETVQRLLPVLCQAHGLTPEQVVAIASHDGGKQALETVQRLLP
VLCQAHGLTPEQVVAIASHDGGKQALETVQRLLPVLCQAHGLTPEQVVAIAS
HDGGKQALETVQRLLPVLCQAHGLTPEQVVAIASNIGGKQALETVQALLPVL
CQAHGLTPQQVVAIASNGGGKQALETVQRLLPVLCQAHGLTPQQVVAIASN
NGGRPALE**SIVAQLSRPDPALAALTNDHLVALACLGGRPALDAVKKGLG**

ND6-TALED Right mitoTALE repeat:

DIADLRTLGYSQQQQEKIKPKVRSTVAQHHEALVGHGFTHAHIVALSQH
PAALGTVAVKYQDMIAALPEATHEAIVGVGKQWSGARALEALLTVAGE
LRGPPLQLDTGQLLKIAKRGGVTAVEAVHAWRNALTGAPLNLTPQQVVA
IASNNGGKQALETVQRLLPVLCQAHGLTPEQVVAIASNIGGKQALETVQALL
PVLCQAHGLTPQQVVAIASNGGGKQALETVQRLLPVLCQAHGLTPQQVVAIA
SNNGGKQALETVQRLLPVLCQAHGLTPQQVVAIASNNGGKQALETVQRLLP
VLCQAHGLTPEQVVAIASHDGGKQALETVQRLLPVLCQAHGLTPQQVVAIAS
NGGGKQALETVQRLLPVLCQAHGLTPEQVVAIASNIGGKQALETVQALLPVL
CQAHGLTPQQVVAIASNGGGKQALETVQRLLPVLCQAHGLTPQQVVAIASN
GGGKQALETVQRLLPVLCQAHGLTPQQVVAIASNNGGRPALE**SIVAQLSRPD**
PALAALTNDHLVALACLGGRPALDAVKKGLG

ND1-TALED/mitoABE Left mitoTALE repeat:

DIADLRTLGYSQQQQEKIKPKVRSTVAQHHEALVGHGFTHAHIVALSQH
PAALGTVAVKYQDMIAALPEATHEAIVGVGKQWSGARALEALLTVAGE
LRGPPLQLDTGQLLKIAKRGGVTAVEAVHAWRNALTGAPLNLTPEQVVAI
ASHDGGKQALETVQALLPVLCQAHGLTPQQVVAIASNGGGKQALETVQRLL
PVLCQAHGLTPQQVVAIASNIGGKQALETVQRLLPVLCQAHGLTPEQVVAIAS
NNGGKQALETVQALLPVLCQAHGLTPEQVVAIASHDGGKQALETVQALLPVL
CQAHGLTPEQVVAIASHDGGKQALETVQALLPVLCQAHGLTPEQVVAIASNG
GGKQALETVQRLLPVLCQAHGLTPEQVVAIASNIGGKQALETVQALLPVLCQ
AHGLTPEQVVAIASNNGGKQALETVQRLLPVLCQAHGLTPEQVVAIASHDGG
KQALETVQALLPVLCQAHGLTPEQVVAIASHDGGKQALETVQALLPVLCQAH
GLTPEQVVAIASNNGGKQALETVQALLPVLCQAHGLTPEQVVAIASNGGGKQ
ALETVQRLLPVLCQAHGLTPQQVVAIASNGGGKQALETVQRLLPVLCQAHGL
TPQQVVAIASNGGGRPALE**SIVAQLSRPDPALAALTNDHLVALACLGGRPA**
LDAVKKGLG

ND1-TALED/mitoABE Right mitoTALE repeat:

DIADLRTLGYSQQQQEKIKPKVRSTVAQHHEALVGHGFTHAHIVALSQH
PAALGTVAVKYQDMIAALPEATHEAIVGVGKQWSGARALEALLTVAGE

LRGPPLQLDTGQLLKIAKRGGVTAVEAVHAWRNALTGAPLNLTPEQVVAI
ASNNGGKQALETVQALLPVLCQAHGLTPQQVVAIASNIGGKQALETVQRLL
PVLCQAHGLTPQQVVAIASNNGGKQALETVQRLLPVLCQAHGLTPEQVVAIAS
NGGGKQALETVQALLPVLCQAHGLTPEQVVAIASNGGGKQALETVQALLPVL
CQAHGLTPEQVVAIASNGGGKQALETVQALLPVLCQAHGLTPEQVVAIASNN
GGKQALETVQRLLPVLCQAHGLTPEQVVAIASNIGGKQALETVQALLPVLCQ
AHGLTPEQVVAIASNGGGKQALETVQRLLPVLCQAHGLTPEQVVAIASNNGG
KQALETVQALLPVLCQAHGLTPEQVVAIASHDGGKQALETVQALLPVLCQAH
GLTPEQVVAIASNGGGKQALETVQALLPVLCQAHGLTPEQVVAIASHDGGKQ
ALETVQRLLPVLCQAHGLTPQQVVAIASNIGGKQALETVQRLLPVLCQAHGLT
PEQVVAIASHDGGKQALETVQALLPVLCQAHGLTPQQVVAIASHDGGKQALE
TVQRLLPVLCQAHGLTPQQVVAIASHDGGKQALETVQRLLPVLCQAHGLTPQ
QVVAIASNGGGRPALE**SIVAQLSRPDPALAALTNDHLVALACLGGRPALDA**
VKKGLG

ATP6 (m.T9185)-TALED Left mitoTALE repeat:

DIADLRTLGYSQQQQEKIKPKVRSTVAQHHEALVGHGFTHAHIVALSQH
PAALGTVAVKYQDMIAALPEATHEAIVGVGKQWSGARALEALLTVAGE
LRGPPLQLDTGQLLKIAKRGGVTAVEAVHAWRNALTGAPLNLTPDQVVA
IASNGGGKQALETVQRLLPVLCQAHGLTPAQVVAIASNGGGKQALETVQRLL
PVLCQAHGLTPAQVVAIASNGGGKQALETVQRLLPVLCQAHGLTPDQVVAIAS
HDGGKQALETVQRLLPVLCQAHGLTPAQVVAIASNIGGKQALETVQRLLPVL
CQAHGLTPDQVVAIASHDGGKQALETVQRLLPVLCQAHGLTPAQVVAIASNIG
GKQALETVQRLLPVLCQAHGLTPDQVVAIASHDGGKQALETVQRLLPVLCQA
HGLTPAQVVAIASNGGGKQALETVQRLLPVLCQAHGLTPDQVVAIASNGGGK
QALETVQRLLPVLCQAHGLTPAQVVAIASHDGGKQALETVQRLLPVLCQAHG
LTPDQVVAIASNGGGKQALETVQRLLPVLCQAHGLTPAQVVAIASNIGGKQAL
ETVQRLLPVLCQAHGLTPDQVVAIASNNGGKQALETVQRLLPVLCQAHGLTP
DQVVAIASNGGGRPALE**SIVAQLSRPDPALAALTNDHLVALACLGGRPALD**
AVKKGLG

ATP6 (m.T9185)-TALED Right mitoTALE repeat:

DIADLRTLGYSQQQQEKIKPKVRSTVAQHHEALVGHGFTHAHIVALSQH
PAALGTVAVKYQDMIAALPEATHEAIVGVGKQWSGARALEALLTVAGE
LRGPPLQLDTGQLLKIAKRGGVTAVEAVHAWRNALTGAPLNLTPDQVVA
IASHDGGKQALETVQRLLPVLCQAHGLTPAQVVAIASNIGGKQALETVQRLL
PVLCQAHGLTPDQVVAIASNGGGKQALETVQRLLPVLCQAHGLTPAQVVAIAS
NGGGKQALETVQRLLPVLCQAHGLTPDQVVAIASNIGGKQALETVQRLLPVL
CQAHGLTPDQVVAIASNGGGKQALETVQRLLPVLCQAHGLTPDQVVAIASNN
GGKQALETVQRLLPVLCQAHGLTPDQVVAIASNGGGKQALETVQRLLPVLCQ
AHGLTPDQVVAIASNNGGKQALETVQRLLPVLCQAHGLTPDQVVAIASNGGG
KQALETVQRLLPVLCQAHGLTPDQVVAIASNGGGKQALETVQRLLPVLCQAH
GLTPAQVVAIASNNGGKQALETVQRLLPVLCQAHGLTPDQVVAIASNGGGKQ
ALETVQRLLPVLCQAHGLTPAQVVAIASHDGGKQALETVQRLLPVLCQAHGL

TPDQVVAIASNNGGKQALETVQRLLPVLCQAHGLTPDQVVAIASNGGGRPAL
E**SIVAQLSRPDPALAALTNDHLVALACLGGRPALDAVKKGLG**

TRNG/TG (m.T10010)-TALED Left mitoTALE repeat:

DIADLRTLGYSQQQQEKIKPKVRSTVAQHHEALVGHGFTHAHIVALSQH
PAALGTVAVKYQDMIAALPEATHEAIVGVGKQWSGARALEALLTVAGE
LRGPPLQLDTGQLLKIAKRGGVTAVEAVHAWRNALTGAPLNLTPDQVVA
IASHDGGKQALETVQRLLPVLCQAHGLTPAQVVAIASNGGGKQALETVQRLL
PVLCQAHGLTPAQVVAIASNGGGKQALETVQRLLPVLCQAHGLTPAQVVAIAS
NIGGKQALETVQRLLPVLCQAHGLTPDQVVAIASHDGGKQALETVQRLLPVL
CQAHGLTPAQVVAIASNGGGKQALETVQRLLPVLCQAHGLTPDQVVAIASHD
GGKQALETVQRLLPVLCQAHGLTPAQVVAIASNGGGKQALETVQRLLPVLCQ
AHGLTPDQVVAIASNGGGKQALETVQRLLPVLCQAHGLTPAQVVAIASNGGG
KQALETVQRLLPVLCQAHGLTPDQVVAIASNGGGKQALETVQRLLPVLCQAH
GLTPAQVVAIASNIGGKQALETVQRLLPVLCQAHGLTPDQVVAIASNNGGKQA
LETVQRLLPVLCQAHGLTPDQVVAIASNGGGKQALETVQRLLPVLCQAHGLT
PDQVVAIASNIGGKQALETVQRLLPVLCQAHGLTPDQVVAIASNGGGRPALE**S**
IVAQLSRPDPALAALTNDHLVALACLGGRPALDAVKKGLG

TRNG/TG (m.T10010)-TALED Right mitoTALE repeat:

DIADLRTLGYSQQQQEKIKPKVRSTVAQHHEALVGHGFTHAHIVALSQH
PAALGTVAVKYQDMIAALPEATHEAIVGVGKQWSGARALEALLTVAGE
LRGPPLQLDTGQLLKIAKRGGVTAVEAVHAWRNALTGAPLNLTPDQVVA
IASNIGGKQALETVQRLLPVLCQAHGLTPDQVVAIASNNGGKQALETVQRLL
PVLCQAHGLTPDQVVAIASNGGGKQALETVQRLLPVLCQAHGLTPDQVVAIA
SNGGGKQALETVQRLLPVLCQAHGLTPDQVVAIASNIGGKQALETVQRLLPV
LCQAHGLTPDQVVAIASNIGGKQALETVQRLLPVLCQAHGLTPDQVVAIASNG
GGKQALETVQRLLPVLCQAHGLTPDQVVAIASNGGGKQALETVQRLLPVLCQ
AHGLTPDQVVAIASNNGGKQALETVQRLLPVLCQAHGLTPDQVVAIASNNGG
KQALETVQRLLPVLCQAHGLTPAQVVAIASNIGGKQALETVQRLLPVLCQAH
GLTPDQVVAIASNIGGKQALETVQRLLPVLCQAHGLTPDQVVAIASNNGGKQ
ALETVQRLLPVLCQAHGLTPDQVVAIASNGGGRPALE**SIVAQLSRPDPALAA**
LTNDHLVALACLGGRPALDAVKKGLG
